# Diagnosing Ring Current(s) in Figure-Eight Skeletons: A 3D Through-Space Conjugation in the Two-Loops Crossing

**DOI:** 10.1021/acs.orglett.2c01625

**Published:** 2022-07-07

**Authors:** Katarzyna Wypych, Maria Dimitrova, Dage Sundholm, Miłosz Pawlicki

**Affiliations:** † Faculty of Chemistry, Jagiellonian University, Gronostajowa 2, 30-387 Kraków, Poland; ‡ Department of Chemistry, University of Wrocław, F. Joliot-Curie 14, 503833 Wrocław, Poland; § Department of Chemistry, University of Helsinki, P.O. Box 55, A. I. Virtasen aukio 1, FIN-00014 Helsinki, Finland

## Abstract

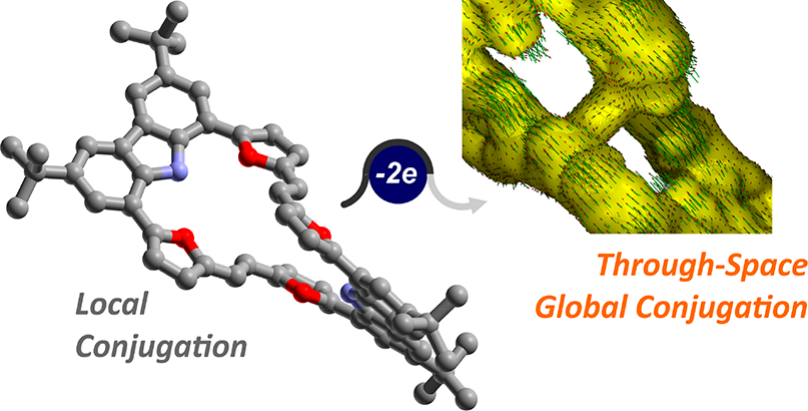

The macrocyclic structures with local conjugation readily undergo a redox-triggered change in the diatropic character, leading to a global current–density pathway of the doubly charged systems. The figure-eight geometry of the neutral dimer does not significantly change upon oxidation according to the spectroscopic and computational data. The oxidation leads to 3D cross-conjugation at the intersection of the two ethylene bridges resulting in a global ring current.

Delocalization of π electrons observed in conjugated systems and usually correlated with aromatic or antiaromatic character is one of the most important properties of cyclic unsaturated molecules. Over the years, the delocalization concept, defined by Hückel as the (4*n*+2) and (4*n*) π-electron rule and easily applicable to the archetypal aromatic (benzene) and antiaromatic (cyclobutadiene) hydrocarbons, respectively, has been deeply modified and substantially extended.^1^ Going beyond the fundamental understanding of the concept originally assigned to planar molecules eventually revealed a complexity of this phenomenon^2^ especially in the spatial organization of three-dimensional systems where the molecule topology substantially influences the final behavior. The trivial topology described as a planar Hückel, double-sided, and single-looped molecule (Figure 1, *L*_k_ = 0) describes a classic model of aromatic and antiaromatic conjugations with a diatropic (clockwise) or paratropic (counterclockwise) current, respectively.^2,3^ The planar Hückel topology shows a global conjugation in 2D molecules with a through-bond transfer observed for the double-sided system^3^ that has shown a redox switching between diatropic and paratropic ring currents in molecules of different sizes and shapes^4,5^ but also for a global conjugation of charged derivatives.^6^ The effects of conjugation in 3D helical structures with a figure-eight arrangement is problematic,^7^ as the imprinted spatial orientation of the twisted Hückel topology (*L*_k_ = 2, Figure 1) implies potential limitations of the through-bond conjugation in double-sided derivatives eventually leading to suppression of the global effect of conjugation as predicted theoretically^8^ but also proven experimentally for the porphyrin nanoring.^1b^ In such structures, the ring current is expected to change its direction from, e.g., clockwise to counterclockwise, while passing from one loop to the other (Figure 1) at the crossing-point and caused by the asymmetry of the system. Nevertheless, the majority of reported helical and infinity shaped molecules were reported as molecules with different levels of conjugation; diatropic (4*n*+2) or paratropic (4*n*) did not show the mentioned suppression,^9^ suggesting the presence of an additional type of conjugation path operating within twisted Hückel topology systems.

**Figure 1:**
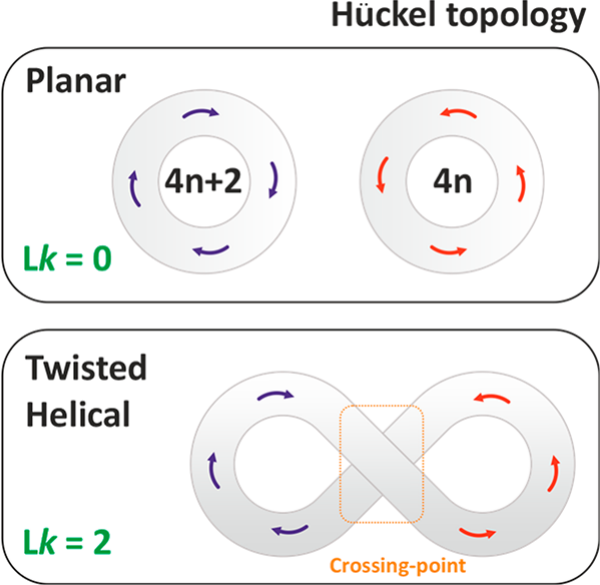
Illustration of independent diatropic and paratropic ring currents and the conflicting local tropicity of a figure eight-shaped molecular structure.

Following those observations, we decided to investigate the conjugation pathways of the figure-eight and redox-switchable molecular structure to explain the ring current(s) behavior in helical systems. As observed, the carbazole- and furan-based macrocycle with *E*-ethylene creates a figure-eight structure with a twisted Hückel topology (*L*_k_ = 2) that when oxidized gives a dication with sustained helicity and a diatropic ring current whose origins were eventually identified as a through-space ring-current pathway at the C–C bridge crossing point. As a reference point for the analysis of the efficiency of ring currents we have involved the *Z*-ethylene-bridged derivative with a Hückel planar topology and very efficient conjugation recorded for an oxidized derivative.

The synthetic approach (Scheme 1) employs a transition-metal-catalyzed process documented as efficient in formation of macrocycles.^10,11^ The incorporated subunits were chosen for being known for forming helical systems (carbazole)^11a^ or being open for redox switching (furan).^6^ The Suzuki–Miyaura coupling performed for **1**(12) and commercially available **2** gave **3** with 67% yield. Compound **3** subjected to the McMurry reaction (Scheme 1) gave two products separated by application of size-exclusion chromatography and assigned as **4** (30%) and **5** (8%) as intramolecular and intermolecular products, respectively.

**Scheme 1:**
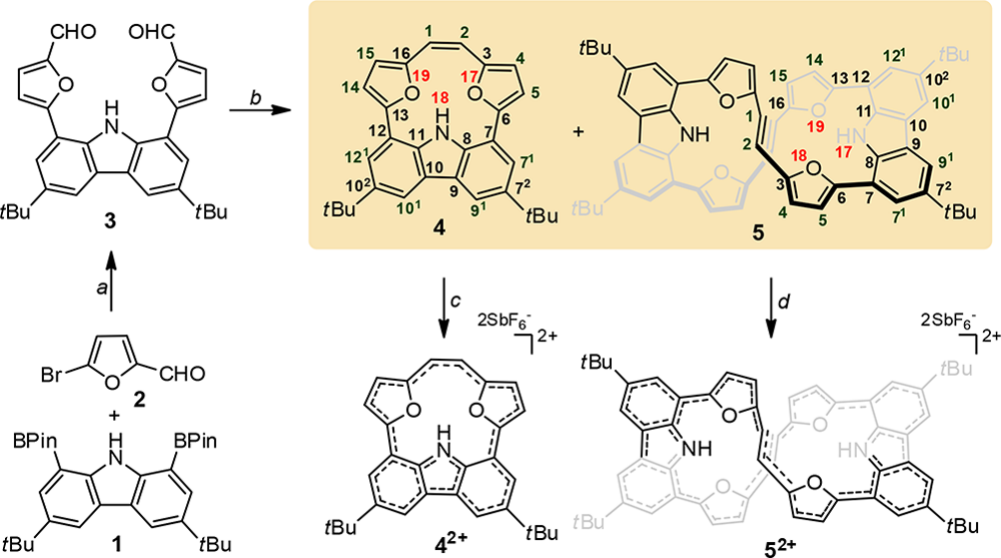
Synthetic Approach for Formation of Macrocycles Conditions: (a) Pd(PPh_3_)_4_, KF, K_2_CO_3_, Tol/DMF, 110 °C, 60–72 h; (b) TiCl_4_, Zn, C_5_H_5_N, dioxane, reflux; (c) 240 K, NOSbF_6_; 200 K, NOSbF_6_.

The formation of both products was confirmed by a monocrystal XRD analysis (Figure 2). Compound **4** shows a steric confinement leading to proximity of the elements constrained inside. The observed distances equaling 2.432(2)/2.433(2) Å for N(18)···O(17)/O(19) and 1.807(2)/1.808(2) Å for H(18)···O(17)/O(19) are shown below the sum of the van der Waals (vdW) radii of nitrogen (hydrogen) and oxygen (N···O 3.07 Å and H···O 2.59 Å),^13^ which in addition forces planarity of the macrocycle (Figure 2A). The X-ray data obtained for **5** (Figure 2B) showed that the observed distances between N(H) and O in both macrocyclic centers are also below the sum of the vdW radii. The *E*-ethylene linkers in **5** are located at the intersection, leading to an almost parallel spatial orientation with an experimental dihedral angle of 15.7°.

**Figure 2:**
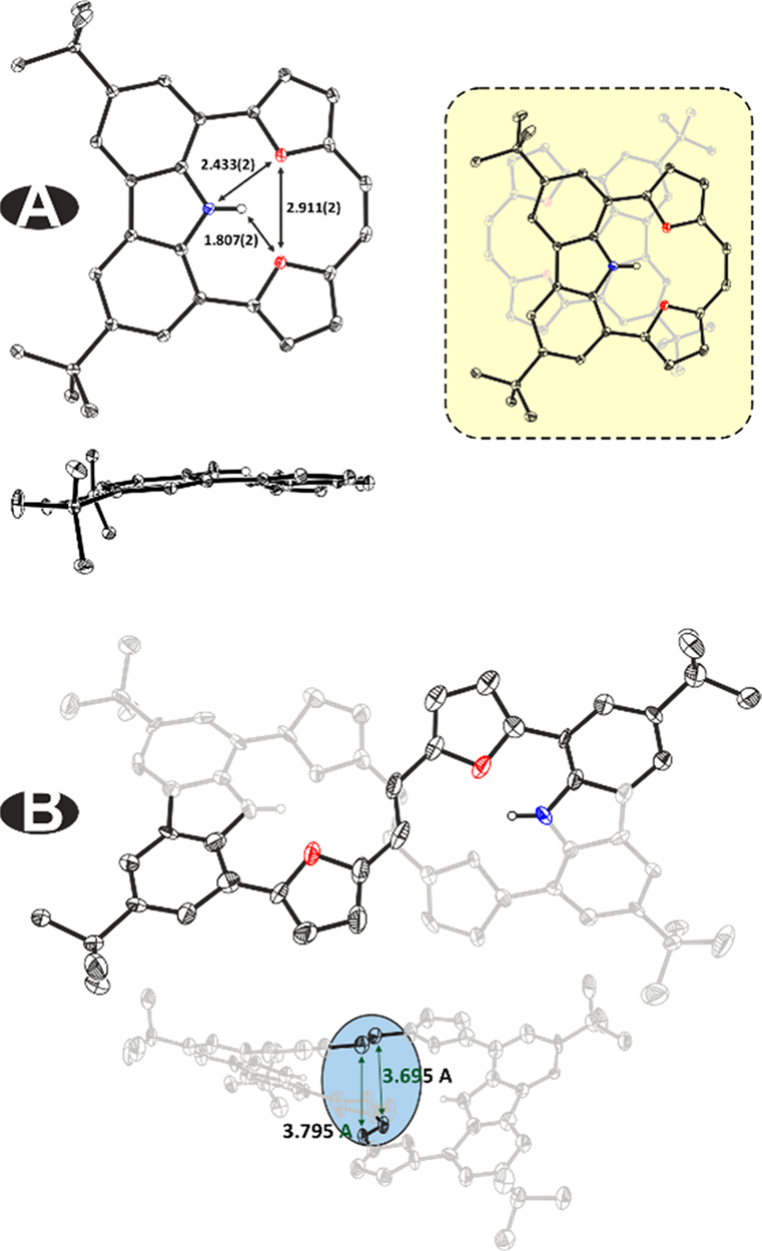
X-ray structures of **4** and **5** (thermal ellipsoids show 50% probability).

Compounds **4** and **5** are locally aromatic according to magnetic criterion,^14^ as documented in the ^1^H NMR spectra (Figure 3A,C) that show rather negligible global delocalization. The signal of the ethylene linker was recorded at δ = 5.88 ppm (**4**) and δ = 6.72 ppm (**5**), and the unsaturated character was further confirmed by the ^13^C chemical shift recorded around 120 ppm. The strength of the N(H)···O interaction observed in the crystal structures is also detected in the ^1^H NMR spectrum, as the chemical shift (δ) of the NH group is substantially downfield shifted in **4** (δ = 15.42 ppm, Figure 3) with a shift of ∼5 ppm with respect to the acyclic **3**. Locally aromatic macrocycles constructed from furan rings have been reported to be redox-switchable, introducing a global diatropic conjugation,^6^ or showed a switching between diatropic and paratropic ring currents.^5^ Following those observations, we have oxidized **4** and **5** with nitrosonium hexafluoroantimonate (NO^+^ SbF_6_^–^) and monitored the product with ^1^H NMR measurements. The oxidation of **4** performed showed a step-by-step process observed in the sequence of quantitative processes **4** → **4**^**•+**^ → **4**^**2+**^ after the first and second equivalent, respectively. Compound **4**^**2+**^ has been identified as reactive at room temperature but stable at 240 K (Scheme 1, path c). All ^1^H NMR resonances were downfield shifted by ∼4 ppm (Figure 3A,B) with respect to **4**, suggesting that **4**^**2+**^ sustains a global diatropic ring current. The oxidation of **5** was performed at low temperature (200 K) to quantitatively form the delocalized derivative **5**^**2+**^ (Scheme 1).

**Figure 3:**
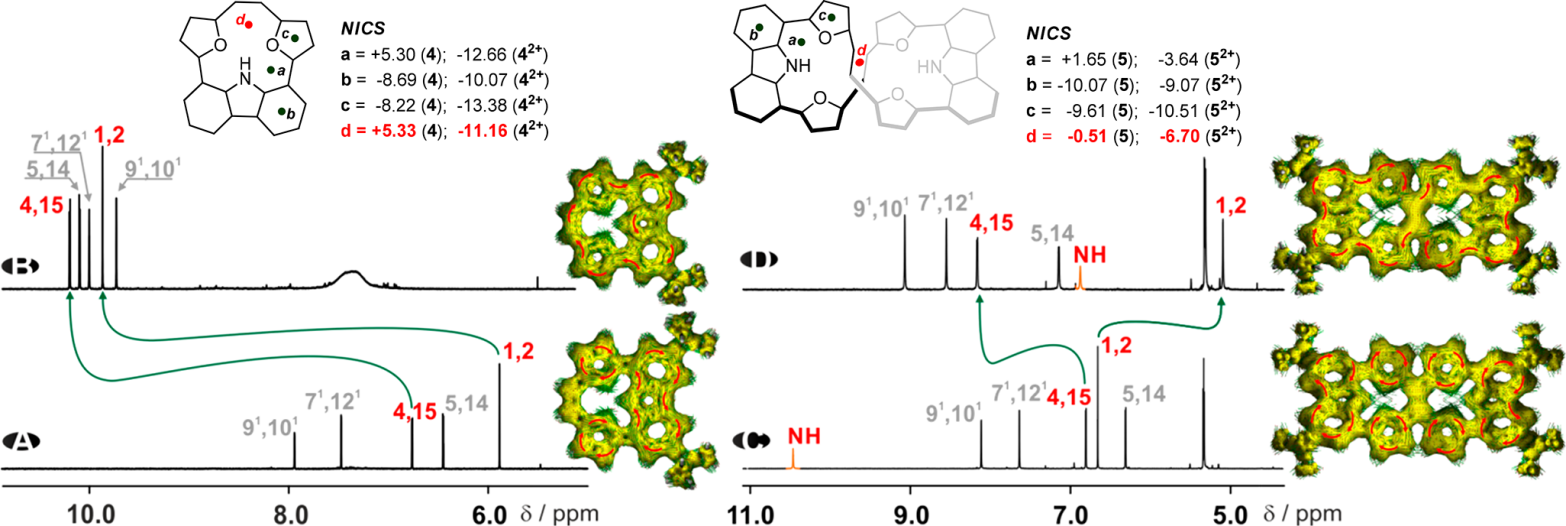
^1^H NMR spectra of **4** (A) and **4**^**2+**^ (B) in acetonitrile-*d*_3_ (500 MHz, 240 K) and **5** (C) and **5**^**2+**^ (D) in dichloromethane-*d*_2_ (500 MHz, 200 K). The AICD presentation of changed local and global currents have been added to each trace (isovalue 0.035); NICS values calculated for selected positions indicate change of local to global delocalization.

The nonplanar geometry of **5** with a figure-eight helical organization confirmed by ^1^H NMR experiments is kept in an oxidized state as judged from the spectroscopic analysis and lack of change in the symmetry of the recorded spectrum (Figure 3). In contrast to the **4**/**4**^**2+**^ couple with a significant downfield relocation of the H(1)/H(2) resonances (from 5.88 (**4**) to 9.86 ppm (**4**^**2+**^)) assigned to C(H)=C(H) positions, in **5**/**5**^**2+**^ the same hydrogens are significantly upfield shifted (from 6.72 (**5**) to 5.10 ppm (**5**^**2+**^) (Figure 3C,D), suggesting that they are influenced by a global diatropic ring current. The shielding effect of the induced global diatropic ring current is seen in the position of the NH group(s). Both resonances are significantly upfield shifted with respect to the starting derivative by Δ ∼11 ppm for **4/4**^**2+**^ pair (from 15.5 to 4 ppm) and by Δ ∼4 ppm for **5/5**^**2+**^ (from 10.8 to 6.95 ppm). A lack of negative chemical shifts in **4**^**2+**^/**5**^**2**^^+^, expected for the strongly shielding effect of the diatropic current, consistently supports the strength of the N(H)···O hydrogen bond that has an opposite influence.^5^ We have employed a theoretical analysis to gain a deeper insight into the observed modulations of the π-conjugation in both redox-switchable couples. The molecular structures of **4**/**4**^2+^ and **5**/**5**^2+^ were fully optimized (see the Supporting Information) starting from the geometry obtained from the X-ray analyses. The optimized structures largely agree with those deduced from the solid-state analyses of **4** and **5** including the separation between the two C2 bridges in **5** (3.695/3.795 Å (X-ray) vs 3.607 Å (calculations), showing that the applied approach reproduces the experimentally available data. Oxidation of **4** yielding **4**^**2+**^ did not significantly change the molecular structure, whereas we found that the separation distance of 3.382 Å at the crossing-point is much shorter in **5**^**2+**^ as compared to the one in **5**. The calculated ^1^H NMR chemical shifts for both couples of **4**/**4**^2+^ and **5**/**5**^2+^ (Table S1) have an excellent correlation with experimental data. The NICS (nucleus independent chemical shifts),^15^ AICD (aniosotropy of the induced current density),^16^ as well as GIMIC (gauge-including magnetically induced currents)^17^ have been used to assess the aromatic character and to determine ring-current strengths. The NICS values obtained for all derivatives and calculated at different points (Figure 3, point d) of the skeletons of the **4** (δ = +5.33 ppm)/**4**^**2+**^ (δ = −11.16 ppm) and **5** (δ = −0.51 ppm)/**5**^**2+**^ (δ = −6.70 ppm) couples consistently support the spectroscopic observations with substantially increased global diatropic delocalization in both dicationic derivatives. The AICD visualization is consistent with the drastic change in delocalization and formation of a global diatropic path in **4**^**2+**^ (Figure 3B) consistent with a conjugation that is substantially different from the local aromatic character of each subunit in **4** (Figure 3A). The AICD analyses have shown a similar behavior documented in the **5**/**5**^**2+**^ couple where the local conjugation in **5** was replaced by the global current observed for **5**^**2+**^ with the clockwise current observed if both loops, but not showing obvious behavior at the C=C bridge crossing (Figure 3D). The more detailed analysis of the AICD plots reveals the presence a through-space conjugation at the crossing point (Figure 4, left) forming a junction for the global diatropic current eventually covering the whole molecule and avoiding the presence of two mutually excluding effects.

**Figure 4:**
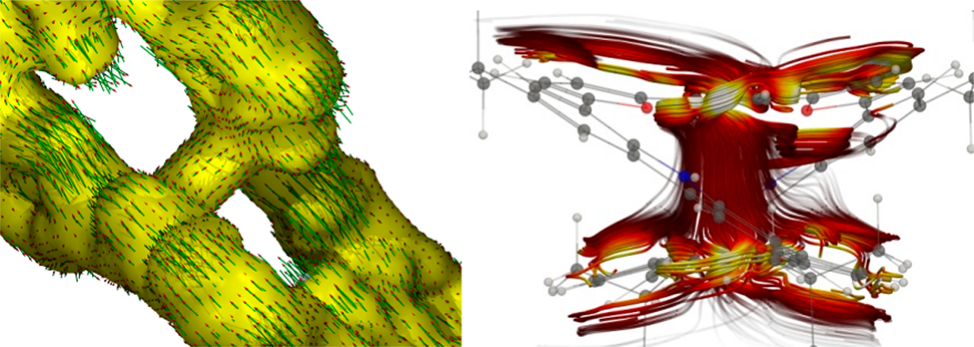
AICD (left, isovalue 0.015) and GIMIC (right) analysis of a through-space conjugation in **5**^**2+**^.

The GIMIC analysis^17^ confirmed that the global effects of conjugation in neutral **4** and **5** are negligible, showing weak paratropic (−3.7 nA/T) and diatropic (0.4 nA/T) ring currents, respectively. The furan and benzene rings sustain local ring currents of about 10 nA/T and a weak ring current of about 1 nA/T flowing around the carbazole moiety. Thus, in both systems, in agreement with the experimental data, a domination of local conjugations of incorporated subunits was observed. The ring-current strengths obtained for the dications **4**^**2+**^ (17.9 nA/T) and **5**^**2+**^ (7.8 nA*/*T) show an efficiency of global conjugation comparable to the archetypal structure of benzene.^18^ In addition, a through-space junction (Figure 4, right) allowing the global effect of conjugation was observed, and the strength of the vertical current-density flux observed on this junction is 6.6 nA/T, which is about half the ring-current strength of benzene.^18^ Thus, the presence of a very effective through-space conjugation path in cationic system **5**^**2+**^ explains the presence of a diatropic conjugation in the helical organization without suppression of the global effect. In contrast to that, **5** shows lack of a through-space current–density flux between the two strands consistent with the spectroscopic observation of the magnetic behavior, at the same time underlining the dependence on the separation of C=C bridges.^19^

In conclusion, the planned and executed research brings a novel point to understanding the fundamental phenomenon of the global delocalization in problematic systems where the opposite tropicities are potentially operating. The ring current(s) in the neutral and cationic helical systems have been analyzed spectroscopically and explained with the support of theoretical predictions. The eventually diagnosed through-space path documented at the crossing point of a cationic system creates an additional and very effective variant of π-electron delocalization with an efficiency comparable to the archetypal motif of benzene. As observed for the **5**/**5**^2+^ couple, the effectiveness of the through-space path depends on the separation of two structural units and increases with closing of the distance. Further experiments to understand this phenomenon are underway in our laboratory.

## Supporting Information Available

General methods, synthesis, and characterization of all compounds; Cartesian coordinates (PDF)
