# Supplementary material for: Diagnosing Ring Current(s) in Figure-Eight Skeletons: A 3D Through-Space Conjugation in the Two-Loops Crossing
Source: Org Lett. 2022 Jul 7;24(27):4876–80. doi: 10.1021/acs.orglett.2c01625 (PMC9348834; doi:10.1021/acs.orglett.2c01625)
Supplement: Supplementary file 1 — ol2c01625_si_001.pdf [file ol2c01625_si_001.pdf]

Diagnosing Ring Current(s) in Figure-Eight Skeletons:
A 3D Through-Space Conjugation in the Two-Loops Crossing

Katarzyna Wypych, Maria Dimitrova, Dage Sundholm and Miłosz Pawlicki*

Contents

1. General Information ... 2
2. Experimental section ... 4
 2.1. Synthetic procedures ... 4
3. NMR spectra ... 5
 3.1. NMR spectra of 3 ... 5
 3.2. NMR spectra of 4. ... 7
 3.3. NMR spectra of 5. ... 10
4. UV-Vis experiments ... 17
5. X-Ray analysis ... 19
6. Theoretical analysis ... 21
 6.1. GIAO data ... 21
 6.2. AICD plots ... 21
 6.3. GIMIC plots ... 23
 6.4. Cartesian coordinates ... 27
  6.4.1. 4 ... 27
  6.4.2. 4^{2+} ... 28
  6.4.3. 5 ... 29
  6.4.4. 5^{2+} ... 30

1. General Information

NMR Spectroscopy. ^{1}H NMR spectra were recorded on a high-field spectrometers (^{1}H 600.15 MHz and 500 MHz, ^{13}C 150 MHz and 125 MHz), equipped with a broadband inverse gradient probehead. Spectra were referenced to the residual solvent signal (chloroform-*d*, 7.24 ppm or dichloromethane-d_2, 5.32 ppm). Two-dimensional NMR spectra were recorded with 2048 data points in the t_2 domain and up to 1024 points in the t_1 domain, with a 1s recovery delay. Structural assignments were made with additional information from gCOSY, gHSQC, and gHMBC experiments.

Mass Spectrometry. High resolution and Accurate Mass spectra were recorded on a Bruker apex ultra FTMS and a Bruker microTOF-Q spectrometers using the electrospray technique.

UV-Vis Spectroscopy. Electronic spectra were recorded on a Varian Carry-50 Bio spectrophotometer. Steady state fluorescence spectra were recorded with a JASCO FP-8600 spectrofluorometer apparatus. Quantum yields were determined using 5,10,15,20-tetraphenylporphyrin (Φ = 0.11 in toluene) as a reference compound.

Theoretical calculations. Geometry optimizations were carried out with the Gaussian 09 software package[1] within unconstrained C1 symmetry, with starting coordinates derived from molecular mechanics or X-ray analysis. The calculations were performed at the density functional theory (DFT) level using Becke's three-parameter exchange functional with the gradient-corrected correlation formula of Lee, Yang and Parr (B3LYP)[2,3] In these calculations, we used the 6-31G(d,p) basis set and PCM solvation model with chloroform as a solvent.[4] Harmonic vibrational frequencies were calculated using analytical second derivatives as a verification of local minimum achievement with no negative frequencies observed. The structures were found to have converged to a minimum on the potential energy. The structure of **5** and **5**$^{2+}$ has been further reoptimized with CAM-B3LYP functional[5] at the same level of theory eventually reproducing the geometry of **5** correlating with the solid-state geometry. Proton chemical shifts were calculated using the GIAO method and referenced to the absolute shielding of tetramethylsilane calculated at the same level of theory.[6] The AICD data was obtained using the same level of theory and IOP(10/93=1) command. The final graphic representation was obtained using the AICD-2.0.0 package supplied by the Authors.

The molecular structures of **5** and **5**$^{2+}$ were also optimized with TURBOMOLE version 7.5[7] at the B3LYP level without considering solvent effects. In the TURBOMOLE calculations a basis set of triple-ζ polarization quality (def2-TZVP)[8], the m5 integration grid[9] and the semiempirical dispersion correction D3(BJ).[10] Also in this approach the obtained geometry restored the spatial organisation observed in the crystal structure. The distance at the crocking point of 5 is 3.607 Å with substantially decreased distance of 3.382 Å for **5**$^{2+}$. The nuclear magnetic shielding tensors were calculated with version 7.5 of the TURBOMOLE program[11,12] at the B3LYP/def2-TZVP level[2,3,8] and the resolution-of-

[1] Frisch, M. J. T., et al. Gaussian 09, Revision E.01; Gaussian, Inc.: Wallingford CT, 2009
[2] Becke, A. D. *J. Chem. Phys.* **1993**, *98*, 5648-5652
[3] Lee, C.; Yang, W.; Parr, R. G. *Phys. Rev. B* **1988**, *37*, 785-789.
[4] Tomasi, J.; Mennucci, B.; Cammi, R. *Chem. Rev.* **2005**, *105*, 2999-3094
[5] T. Yanai, D. P. Tew, N. C. Handy, *Chem. Phys. Lett.*, **2004**, *393*, 51-57,
[6] K. Wolinski, J. F. Hilton, and P. Pulay, *J. Am. Chem. Soc.*, 1990, **112, 8**251-8260
[7] Ahlrichs, R.; Bär, M.; Häser, M.; Horn, H.; Kölmel, C. *Chem. Phys. Lett.* **1989**, *162*, 165-169; http://www.turbomole.com.
[8] Weigend, F.; Ahlrichs, R. *Phys. Chem. Chem. Phys.* **2005**, *7*, 3297-3305.
[9] Treutler, O.; Ahlrichs, R. *J. Chem. Phys.* **1995**, *102*, 346-354
[10] Grimme, S.; Antony, J.; Ehrlich, S.; Krieg, H. *J. Chem. Phys.* **2010**, *132*, 154104
[11] Reiter, K.; Mack, F.; Weigend, F. *J. Chem. Theory Comput.* **2018**, *14*, 191-197
[12] Reiter, K.; Weigend, F.; Wirz, L. N.; Dimitrova, M.; Sundholm, D. *J. Phys. Chem. C* **2019**, *123*,15354-15365.

identity approximation[13] using the optimized molecular structure. The gauge-including magnetically induced current (GIMIC) method[14] was employed to investigate the current-density susceptibility using an open-source program package[15] which takes as input the density matrix and the three magnetically perturbed density matrices that are obtained in calculations of nuclear magnetic shielding tensors using various quantum chemistry packages.

X-Ray Crystallography. X-Ray quality crystals were prepared by slow diffusion of methanol into the CH_2Cl_2 or $CHCl_3$ solution of proper compound. Data were collected at 100K on an Xcalibur PX-κ geometry diffractometer, with Cu Kα radiation (λ=1.5407) or Mo Kα (λ = 0.71073). Data were corrected for Lorentz and polarization effect. The structures were solved by intrinsic phases with SHELXT algorithm implemented into Shelx-2015 package.[16] The refinement of all structures was performed by full matrix least-squares method with using SHELXL algorithm from Shelx-2015 with anisotropic thermal parameters for the non-H atoms Scattering factors were those incorporated in SHELXT (2015). The Olex^2 interface[17] has been used for handling (solving, refining, CIF preparing) all the data. For disordered solvent space in crystal cell the Masks subroutine in OlexSys (equivalent of SQEEZE in Platon) was used.

[13] Eichkorn, K.; Treutler, O.; Öhm, H.; Häser, M.; Ahlrichs, R. *Chem. Phys. Lett.* **1995**, *240*, 283-290
[14] Jusélius, J.; Sundholm, D.; Gauss, J. *J. Chem. Phys.* **2004**, *121*, 3952-3963;
[15] gimic, version 2.0, a current density program, is available at: https://github.com/qmcurrents/gimic. 2019
[16] Sheldrick, G.M. (**2015**). *Acta Cryst. A71*, 3-8; Sheldrick, G.M. (**2015**). *Acta Cryst.* C71, 3-8.
[17] Dolomanov, O.V., Bourhis, L.J., Gildea, R.J, Howard, J.A.K. & Puschmann, H. (**2009**), *J. Appl. Cryst. 42*, 339-341

2. Experimental section

All solvents (MeOH, Ethyl Acetate, $CHCl_3$, n-hexane, acetone, water, dioxane) if not indicated differently were used without purification.CH_2Cl_2 was distilled over CaH_2.THF, toluene, DMF was dried by passing through a silica column with MBraun drying system. Chloroform-*d* was prepared directly before using by passing through a basic alumina column. Dichloromethane-d_2 was used directly. All known compounds (**1**) were synthesized as preciously described.[18] All commercially available reagents (furan derivative, palladium catalyst, phosphines, $TiCl_4$) were used as received.

2.1. Synthetic procedures

Suzuki Coupling

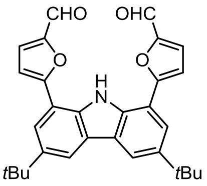

Synthesis of 3. The 250mL two-neck round bottom flask was charged with 3,6-di-tert-butyl-1,8-bis(4,4,5,5-tetramethyl-1,3,2-dioxaborolan-2-yl)-9H-carbazole (2.50 g, 4.7 mmol), tetrakis-(triphenylphosphine) palladium(0) (0.55 g, 0.47 mmol), potassium fluoride (0.97 g, 9.6 mmol), potassium carbonate (1.99 g, 16.8 mmol) and 5-bromo-2-furalaldehyde (1.68 g, 9.6 mmol). The mixture was dried under vacuum for 1h. Then 100 mL of dry toluene and 100 mL of dry DMF were added via syringe. Then the mixture was stirred in 110°C with oil bath overnight. After cooling to room temperature the solution was evaporated in vacuo and then diluted with ethyl acetate and passed through pad of silica gel. After evaporation to dryness product was purified by recrystallization from CH_2Cl_2/MeOH. Yield: 1.47g, 67% **^{1}H NMR** (500 MHz, 300K, $CDCl_3$) δ (ppm) 10.21 (s, 1H), 9.86 (s, 2H), 8.17 (s, ^4J = 1.7 Hz, 2H), 7.87 (s, ^4J = 1.7Hz, 2H), 7.48 (d, ^3J = 3.7 Hz, 2H), 7.12 (d, ^3J = 3.7 Hz, 2H), 1.50 (s, 18H). **^{13}C NMR** (125 MHz, $CDCl_3$) δ 177.6, 159.3, 152.1, 143.2, 134.6, 124.5, 121.4, 121.4, 118.7, 112.0, 108.01, 34.8, 32.0 **UV-Vis** (CH_2Cl_2, 298K, λ) 332, 402 **HRMS** (m/z) found: 490.20 $[M+Na]^+$ (expected 490.20 calcd for $C_{30}H_{29}O_4N$).

McMurry Coupling procedure of 4 and 5: Zinc (2.09 g, 32.1 mmol) and pyridine (12.5µL, 0.0123 g, 0.161 mmol) were added to 120 ml of freshly distilled dioxane, then titanium (IV) chloride (1.75mL, 3.04 g, 16.0 mmol) was added, the resulting mixture was heated with oil bath for about 45 min. After this time **3** (0.75 g, 1.6 mmol) dissolved in dioxane was slowly added and the reaction was heated with oil bath for another 1.5 h protected from light. A saturated ammonium chloride solution was added to the reaction and extracted with water and dichloromethane, and then dried with sodium sulfate. The compound was applied to the SEC column to separate the dimer from the monomer. The first fraction was dimer (**5**) which required additional separation by preparative TLC using hexane:dichloromethane (5:1) as an eluent, it was isolated as the first (yellow) fraction. Yield of compound **5**: 0.12 g, 8.4%. Monomer (**4**) was purified by recrystallization from dichloromethane/hexane solvents to give red crystals. Yield of compound **4**: 0.24 g, 32%.

4: **^{1}H NMR (500 MHz, 300K, $CDCl_3$)** δ (ppm) 15.44 (s, 1H), 7.95 (s, ^4J = 1.4 Hz, 2H), 7.48 (s, ^4J = 1.4 Hz, 2H), 6.77 (d, ^3J = 3.4 Hz, 2H), 6.46 (d, ^3J = 3.7 Hz, 2H), 5.92 (d, 2H) 1.41 (s, 18H). **^{13}C NMR** (150 MHz, $CDCl_3$) δ 155.6, 150.7, 142.9, 139.3, 123.7, 117.7, 117.6, 115.6, 114.0, 111.9, 107.6, 35.0, 32.2; UV-Vis 406, 479 **HRMS (m/z):** 435.2197 $[M+H]^+$ (calcd for $C_{30}H_{29}O_2N$ 435.2193).

5: **^{1}H NMR (500 MHz, 300K, $CDCl_3$)** δ (ppm) 10.46 (s, 1H), 8.10 (s, ^4J = 1.7 Hz, 2H), 7.62 (s, ^4J = 1.7 Hz, 2H), 6.73 (d, ^3J = 3.3 Hz, 2H), 6.24 (d, ^3J = 3.3 Hz, 2H), 6.65 (d, 2H) 1.43 (s, 18H). **^{13}C NMR** (150 MHz, $CDCl_3$) δ 153.6, 152.3 142.6, 134.9, 123.6, 119.5, 116.9, 114.4, 113.8, 111.3, 108.2, 34.9, 32.1; **UV-Vis** 336, 357, 433 **HRMS (m/z):** 870.4391 $[M+H]^+$ (calcd for $C_{60}H_{58}N_2O_4$ 870.4391).

[18] Ch. Maeda, T. Tokada, T. Ema *Org. Lett.* **2015**, *17*, 3090-3093

3. NMR spectra

3.1. NMR spectra of 3

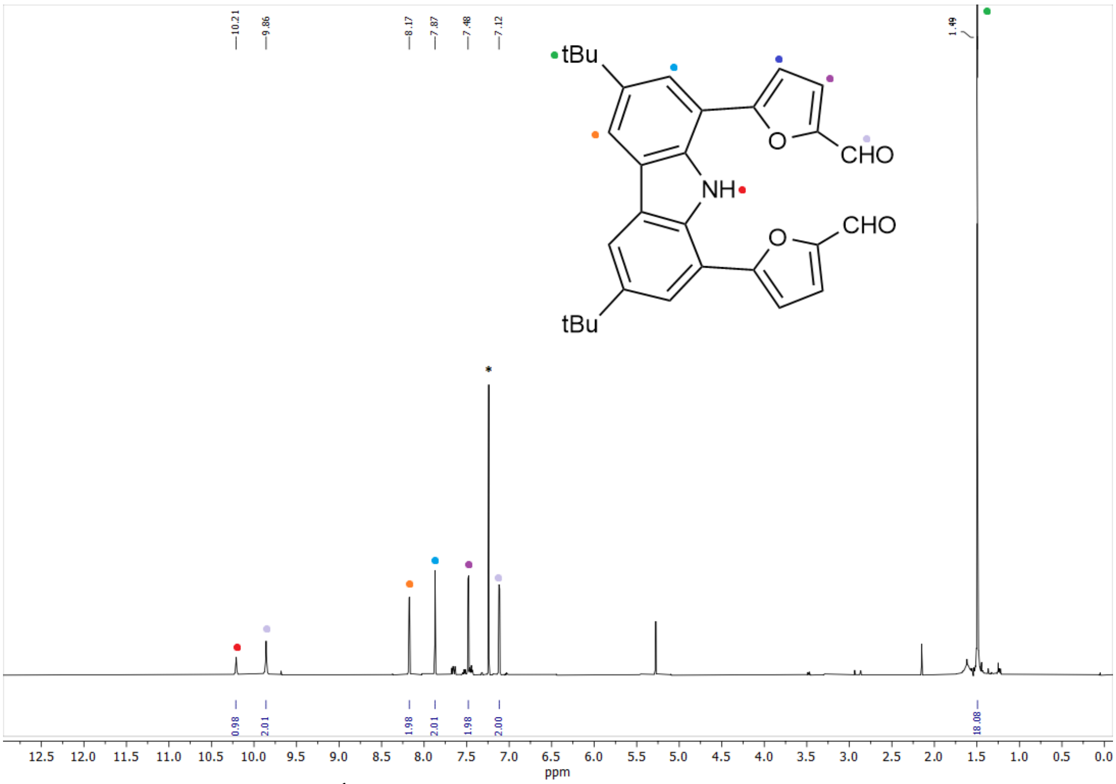

Figure S1. ^{1}H NMR spectrum of **3** (600 MHz, $CDCl_3$, 300 K)

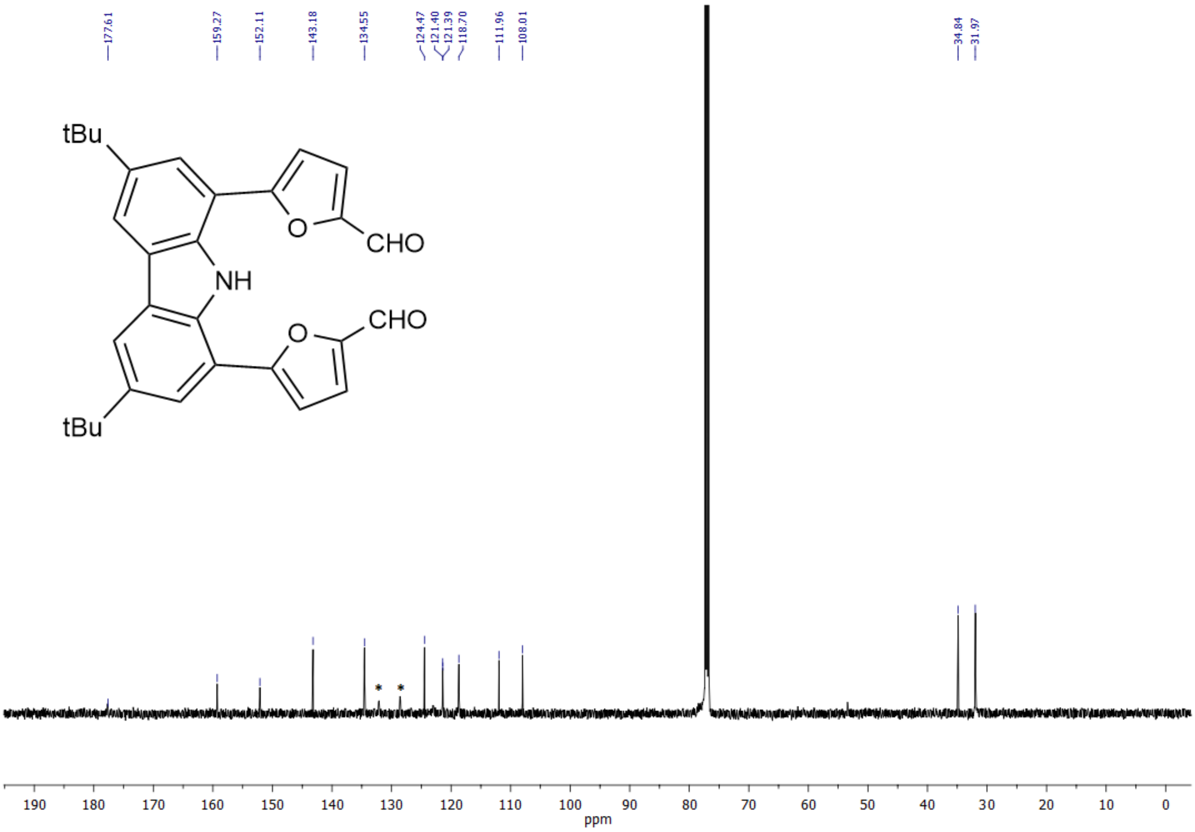

Figure S2. ^{13}C NMR spectrum of **3** (150 MHz, $CDCl_3$, 300 K).

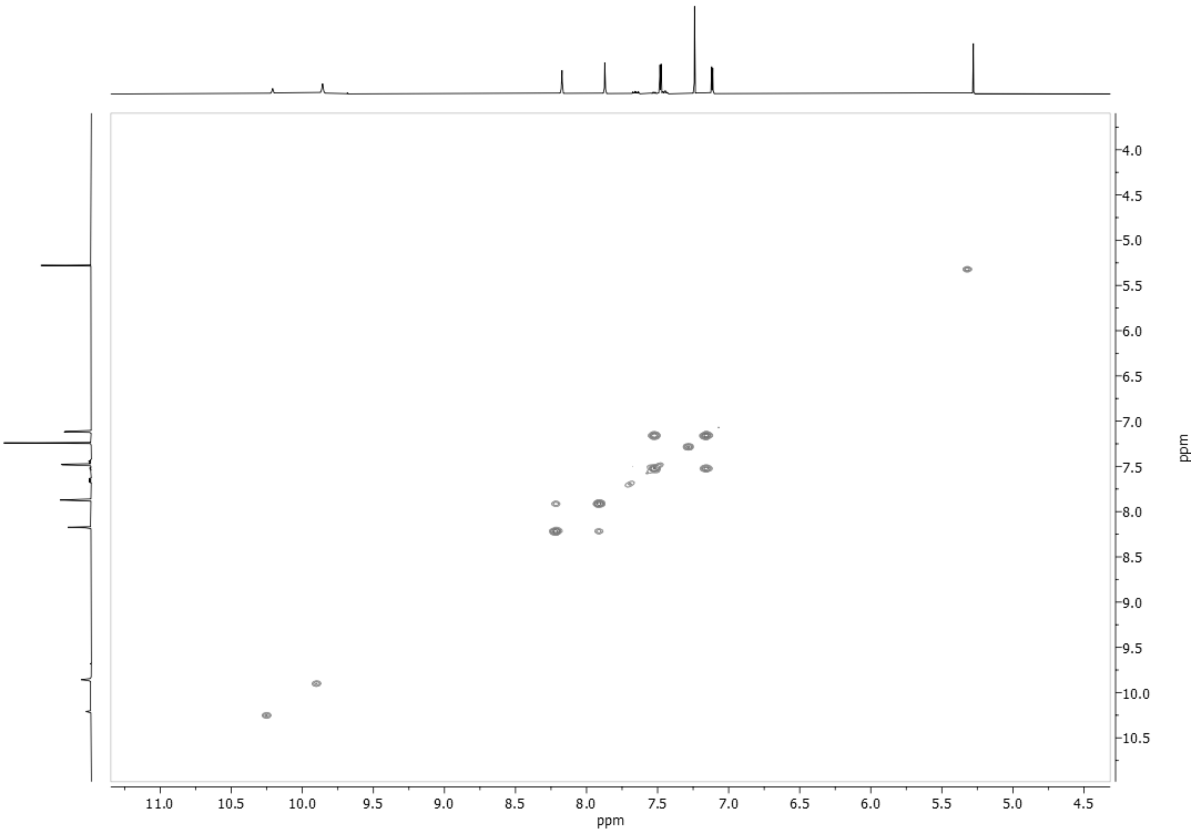

Figure S3. ^{1}H-^{1}H COSY NMR spectrum of **3** (600 MHz, $CDCl_3$, 300 K)

3.2. NMR spectra of 4.

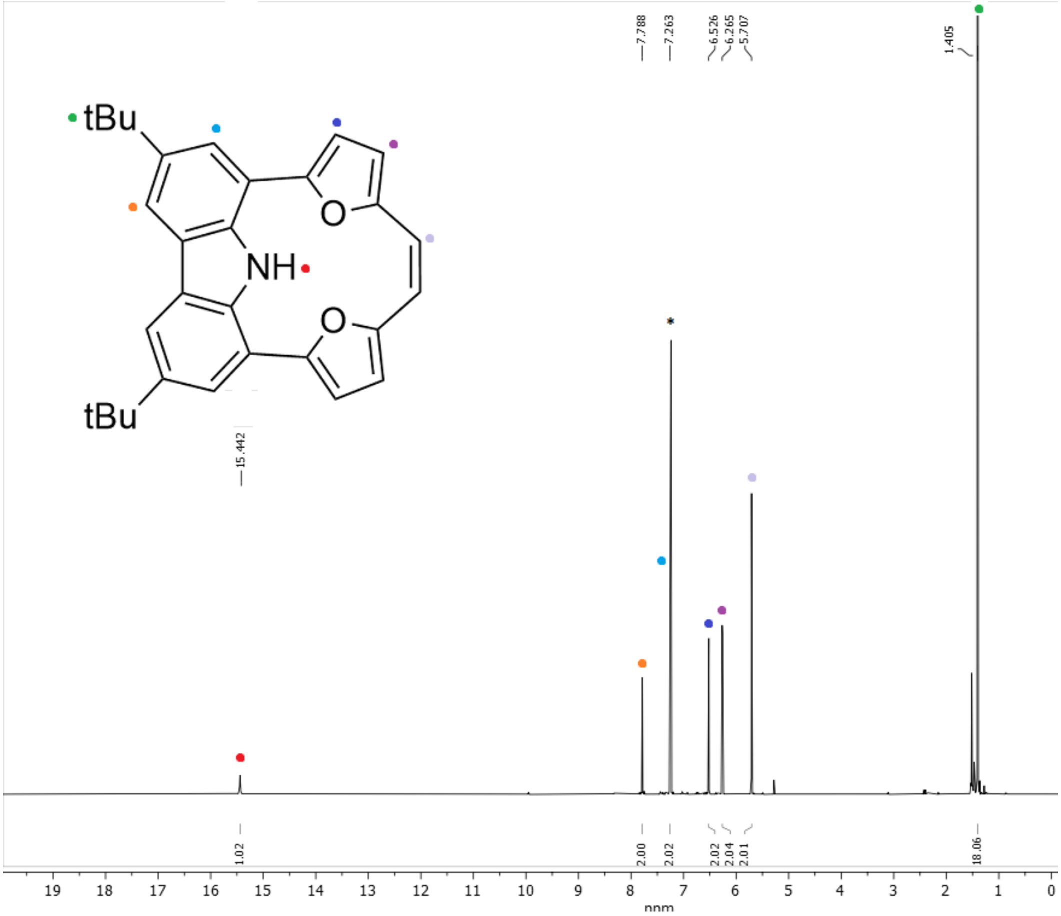

Figure S4. ^{1}H NMR spectrum of **4** (600 MHz, $CDCl_3$, 300 K)

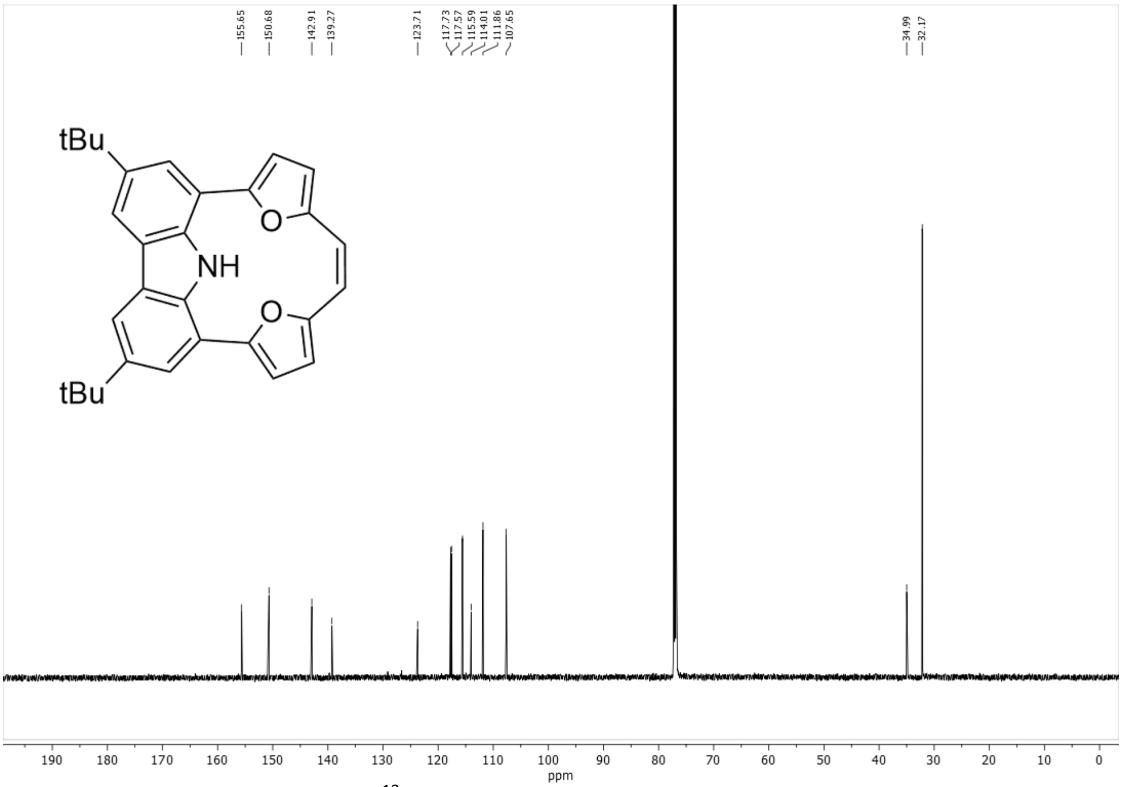

Figure S5. ^{13}C NMR spectrum of **4** (150 MHz, $CDCl_3$, 300 K).

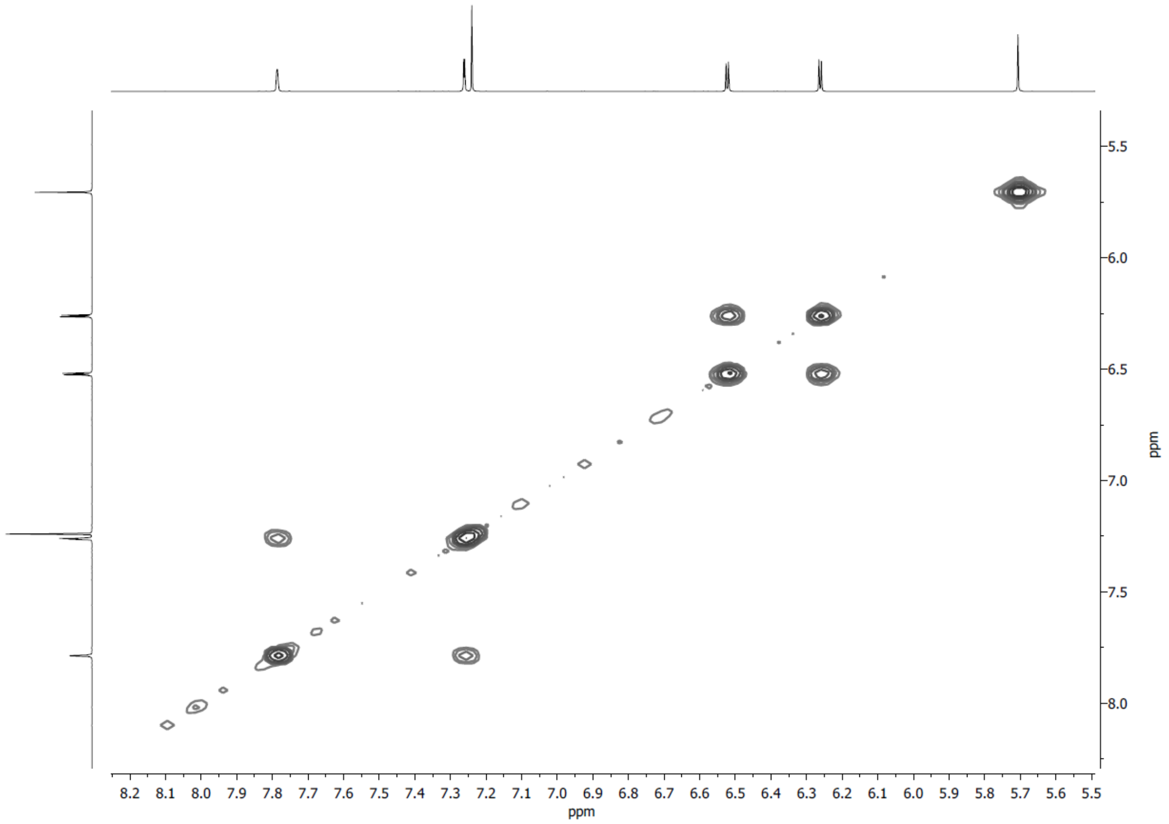

Figure S6. ^{1}H-^{1}H COSY NMR spectrum of **4** ($CDCl_3$, 500 MHz, 298 K)

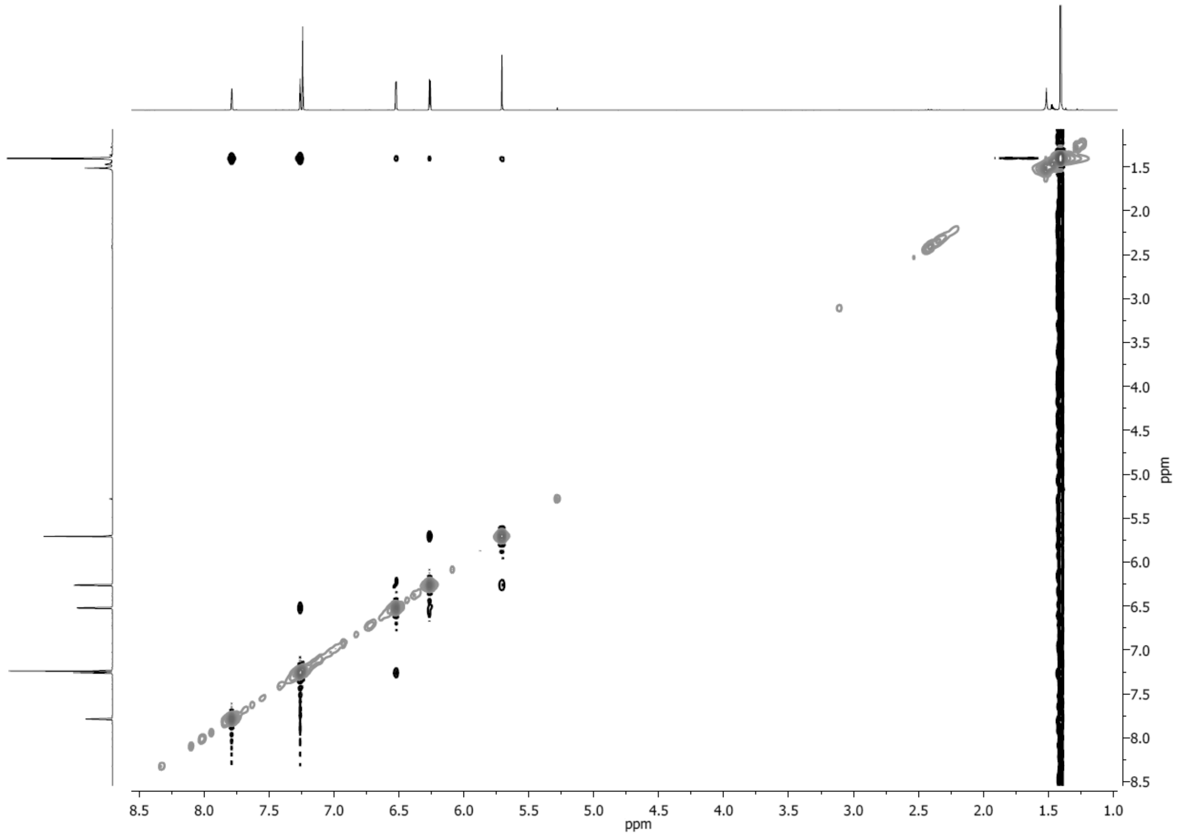

Figure S7. ^{1}H-^{1}H NOESY spectrum of **4** ($CDCl_3$, 600 MHz, 298 K)

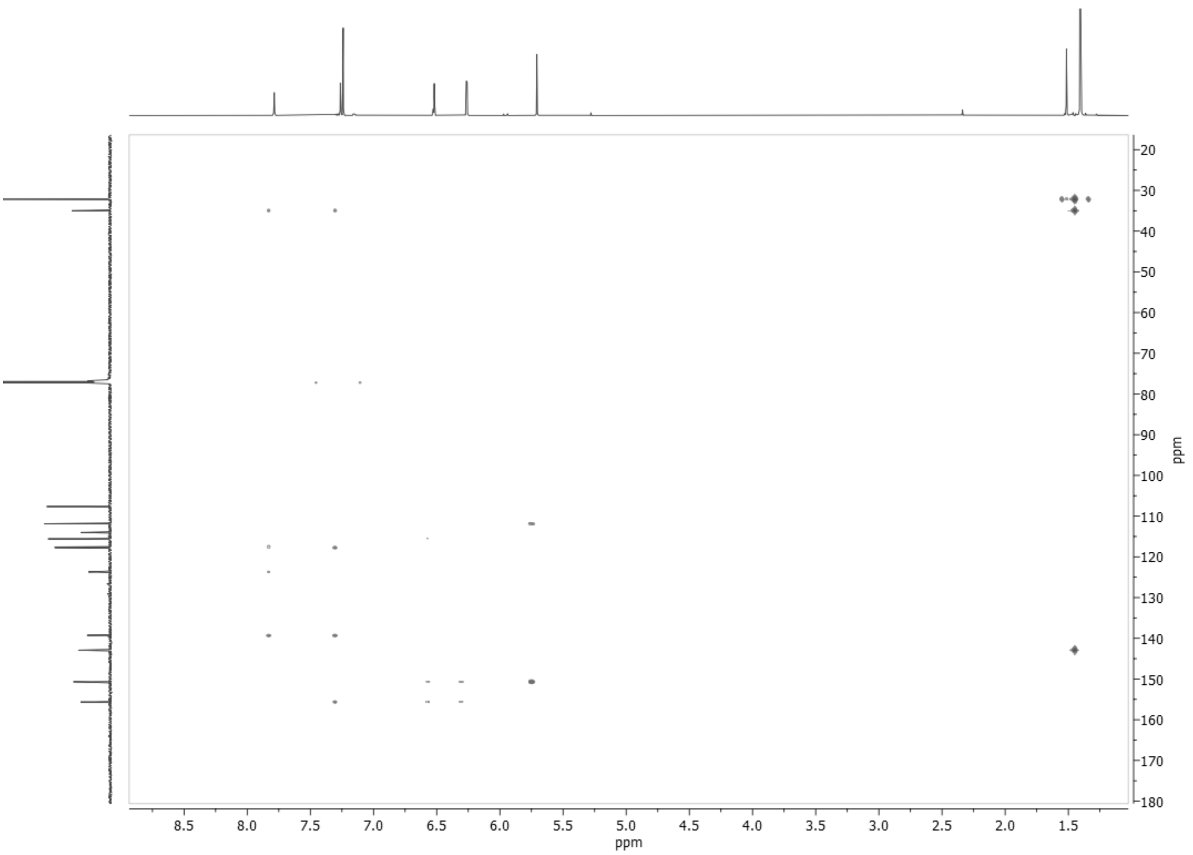

Figure S8. ^{1}H-^{13}C HMBC NMR spectrum of **4** ($CDCl_3$, 600 MHz, 298 K)

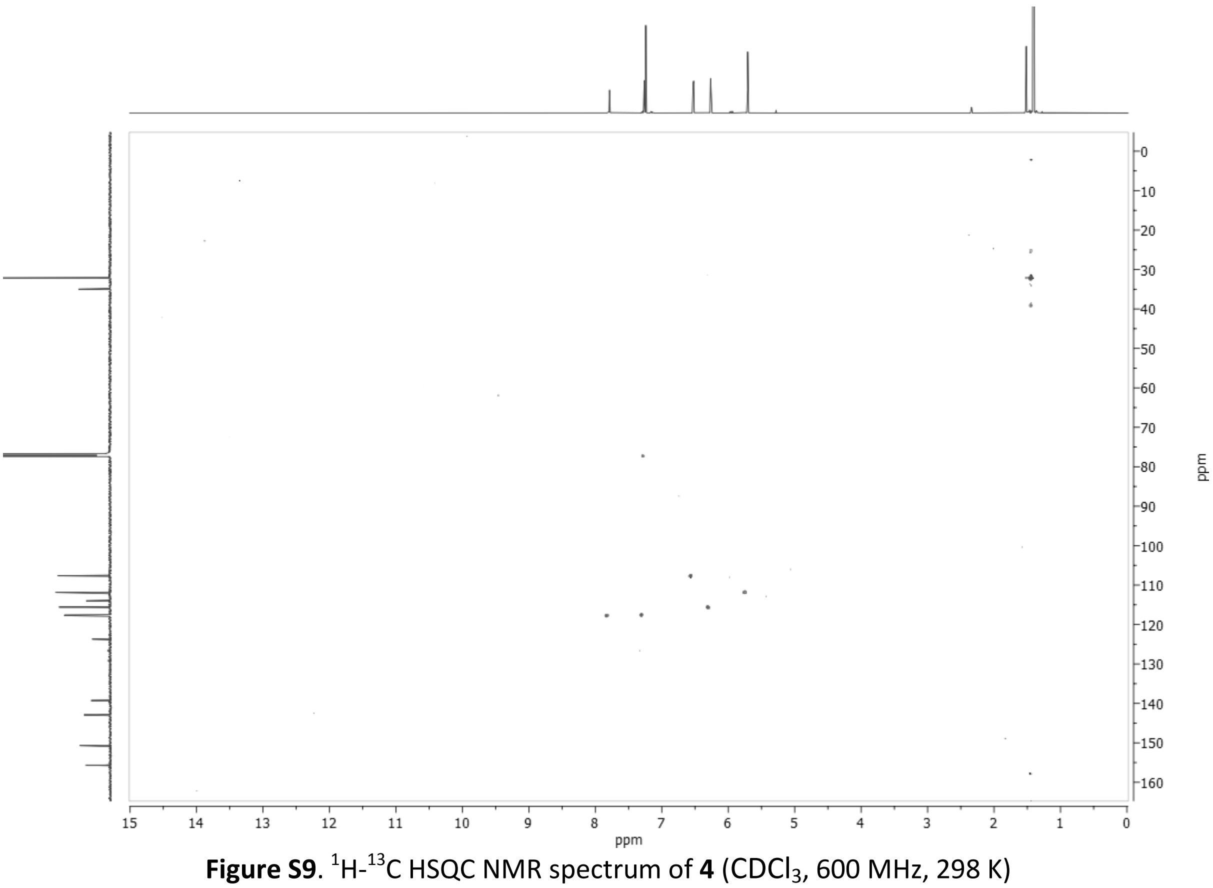

Figure S9. ^{1}H-^{13}C HSQC NMR spectrum of **4** ($CDCl_3$, 600 MHz, 298 K)

3.3. NMR spectra of 5.

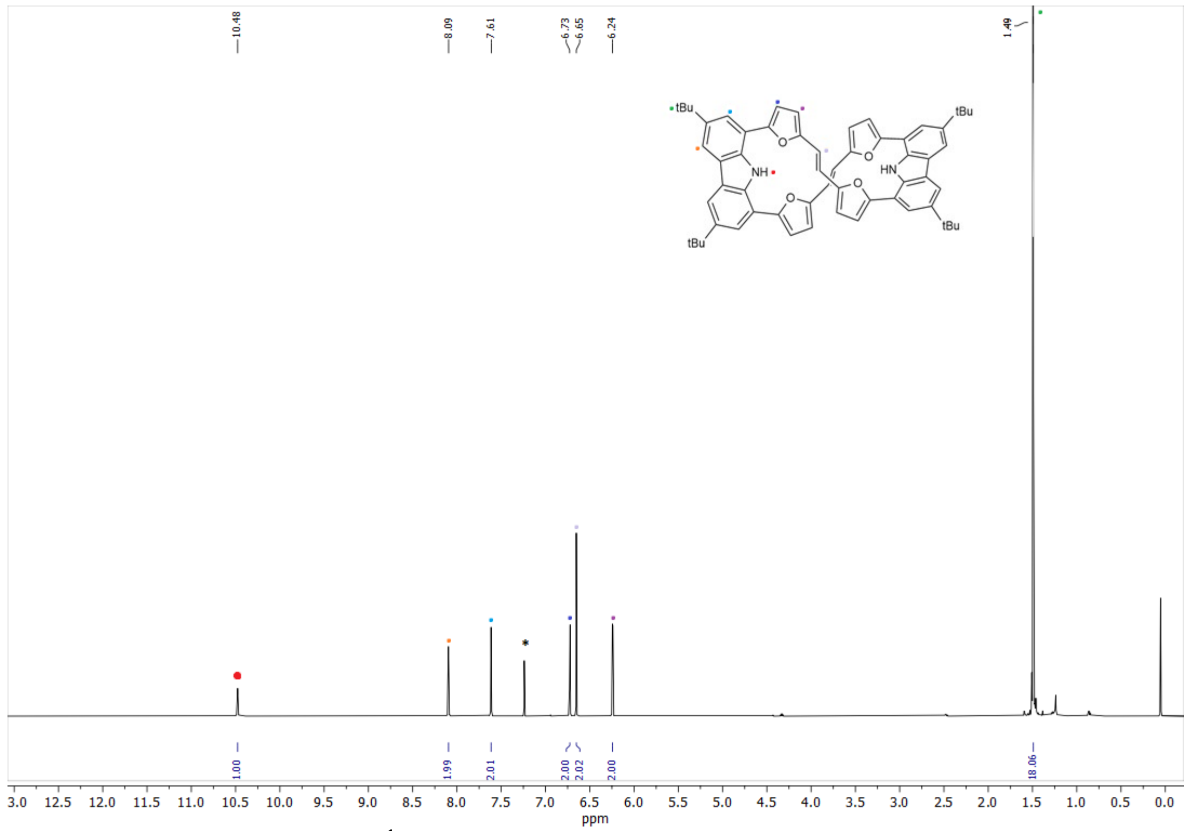

Figure S10. ^{1}H NMR spectrum of **5** (600 MHz, $CDCl_3$, 300 K)

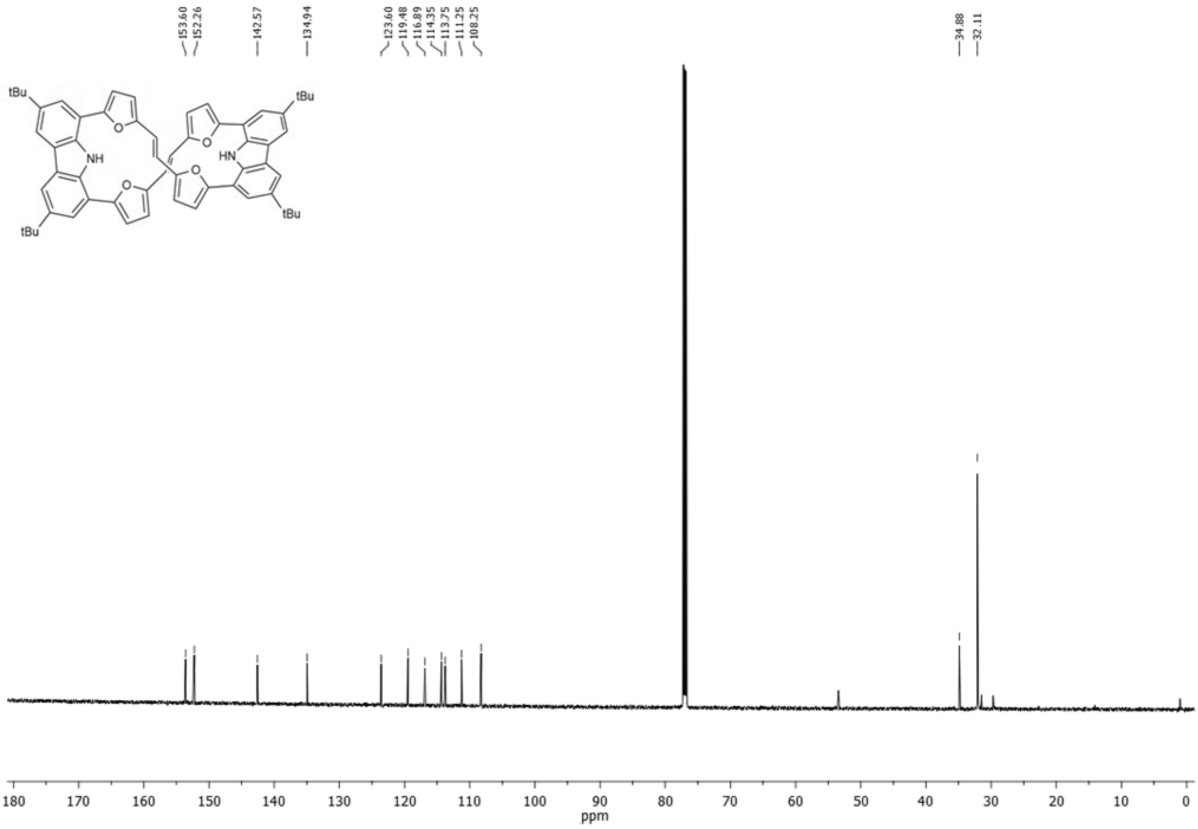

Figure S11. ^{13}C NMR spectrum of **5** (150 MHz, $CDCl_3$, 300 K).

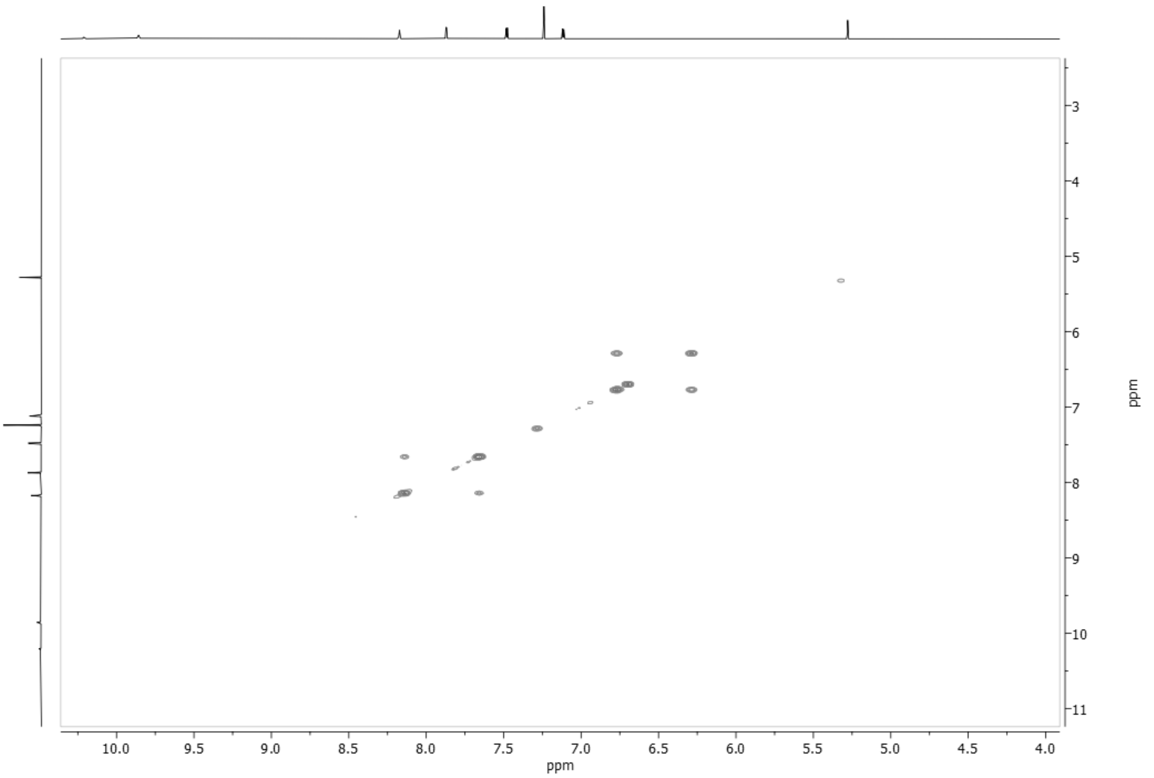

Figure S12. ^{1}H-^{1}H COSY NMR spectrum of **5** ($CDCl_3$, 500 MHz, 298 K)

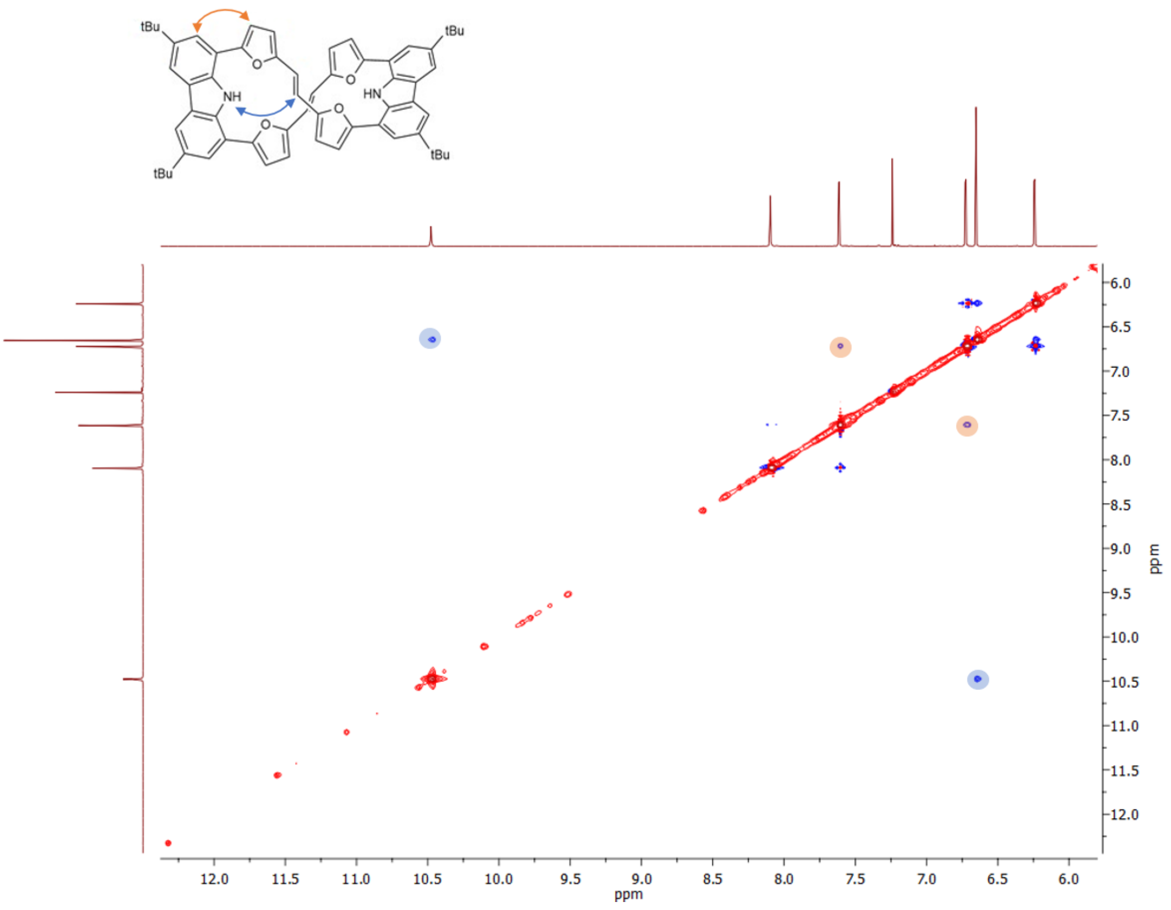

Figure S13. ^{1}H-^{1}H NOESY spectrum of **5** ($CDCl_3$, 600 MHz, 298 K)

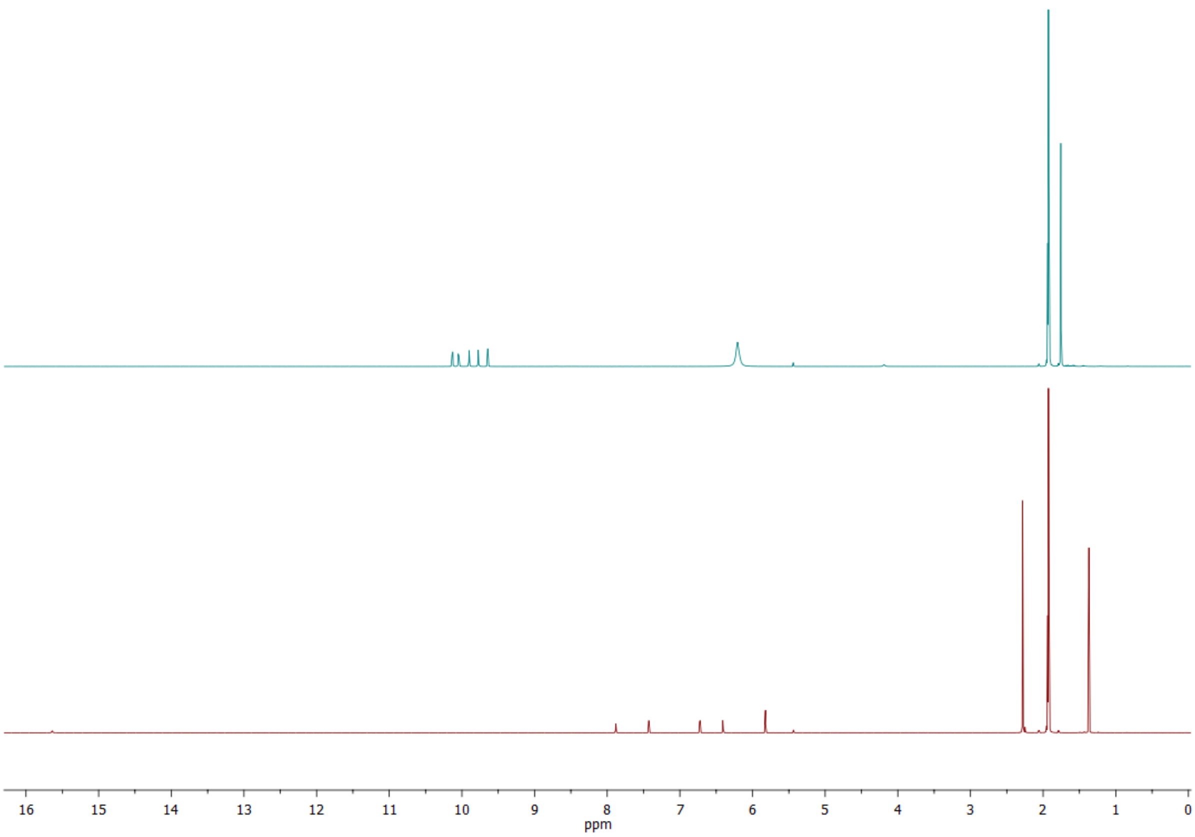

Fig. S14. ^{1}H NMR-monitored titration of **4** and **4^{2+}** with $NOSbF_6$ (acetonitrile-d_3, 500 MHz, 230 K); red – without; cyan – 2eq

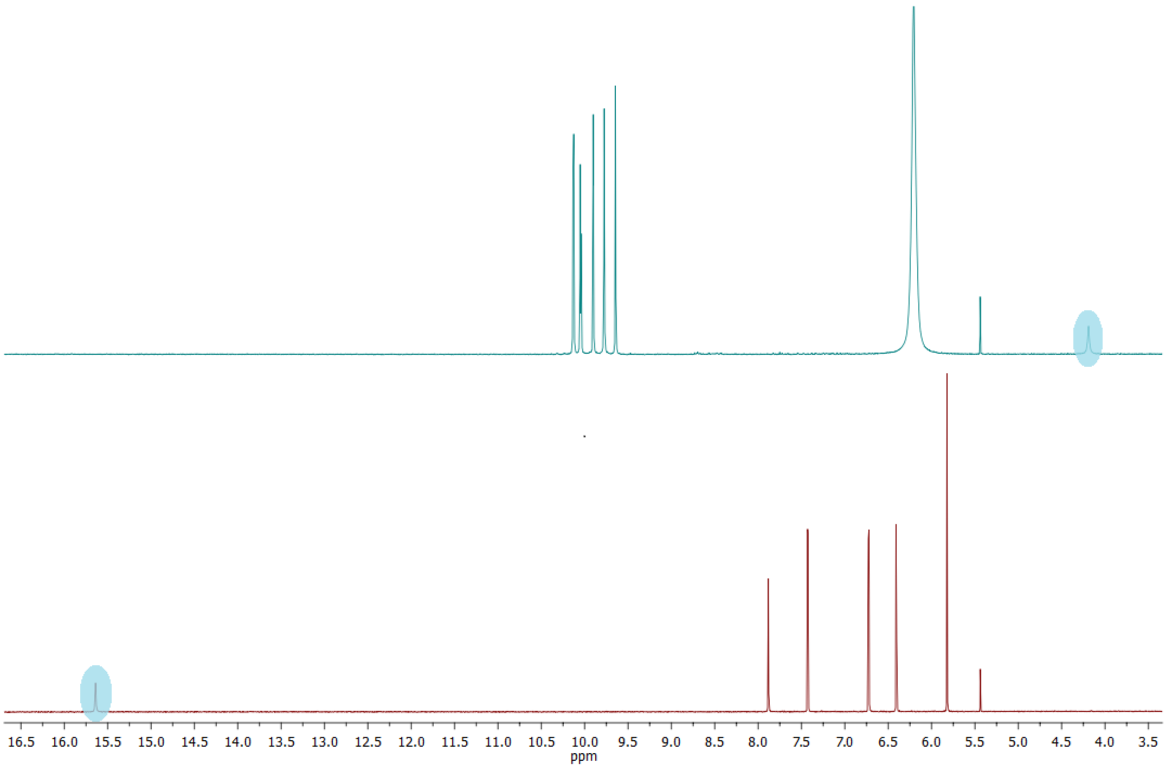

Fig. S15. Zoom on aromatic region ^{1}H NMR-monitored titration of **4** and **4^{2+}** with $NOSbF_6$ (acetonitrile-d_3, 500 MHz, 230 K); red – without; cyan – 2eq

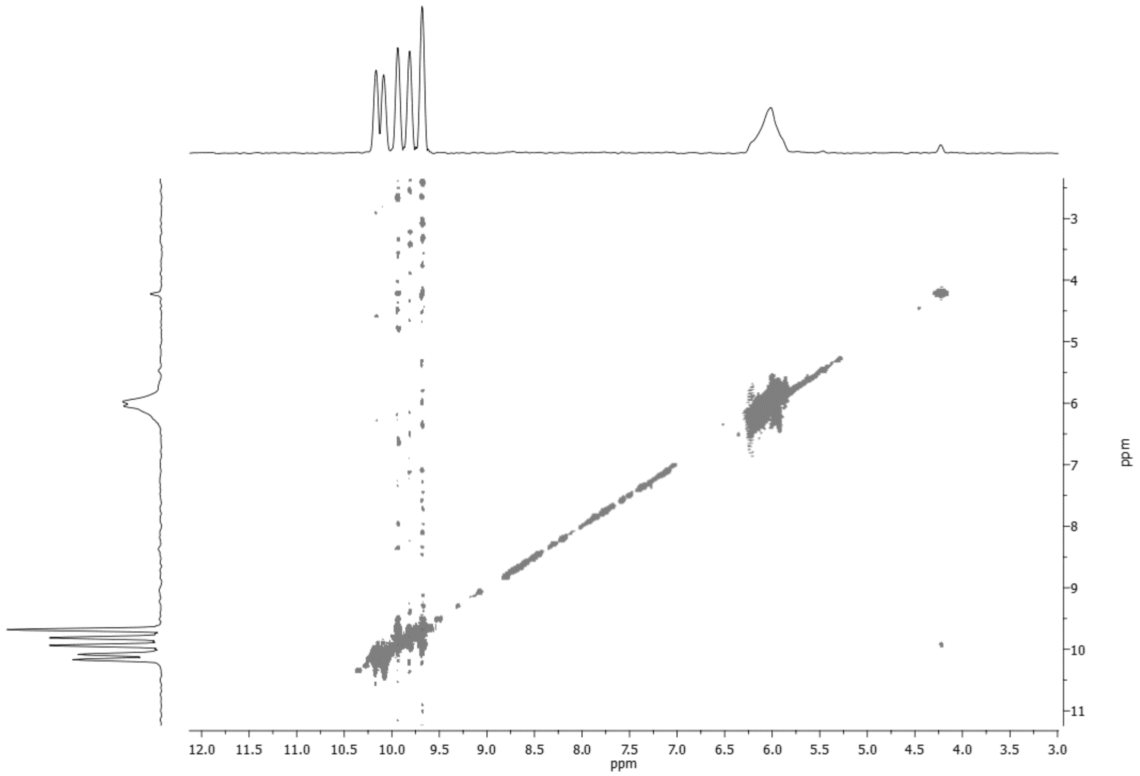

Figure S16. ^{1}H-^{1}H COSY NMR spectrum (zoom aromatic region) of **4**$^{2+}$ (acetonitrile-d_3, 500 MHz, 230 K)

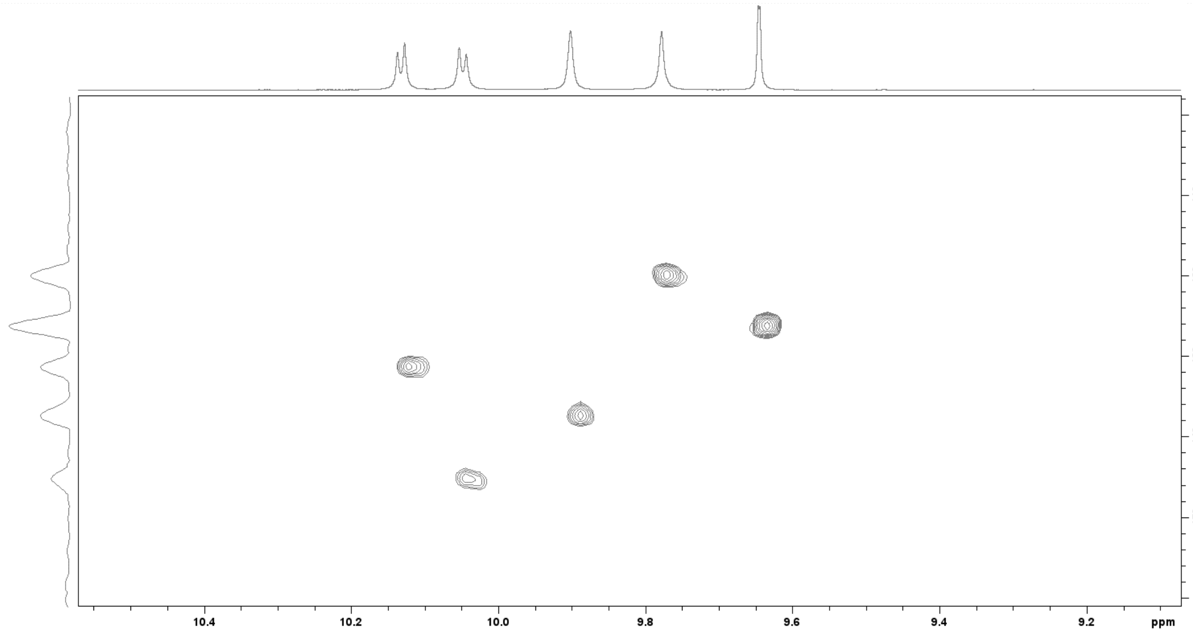

Figure S17. ^{1}H-^{13}C HSQC NMR spectrum of **4**$^{2+}$ (acetonitrile-d_3, 500 MHz, 230 K)

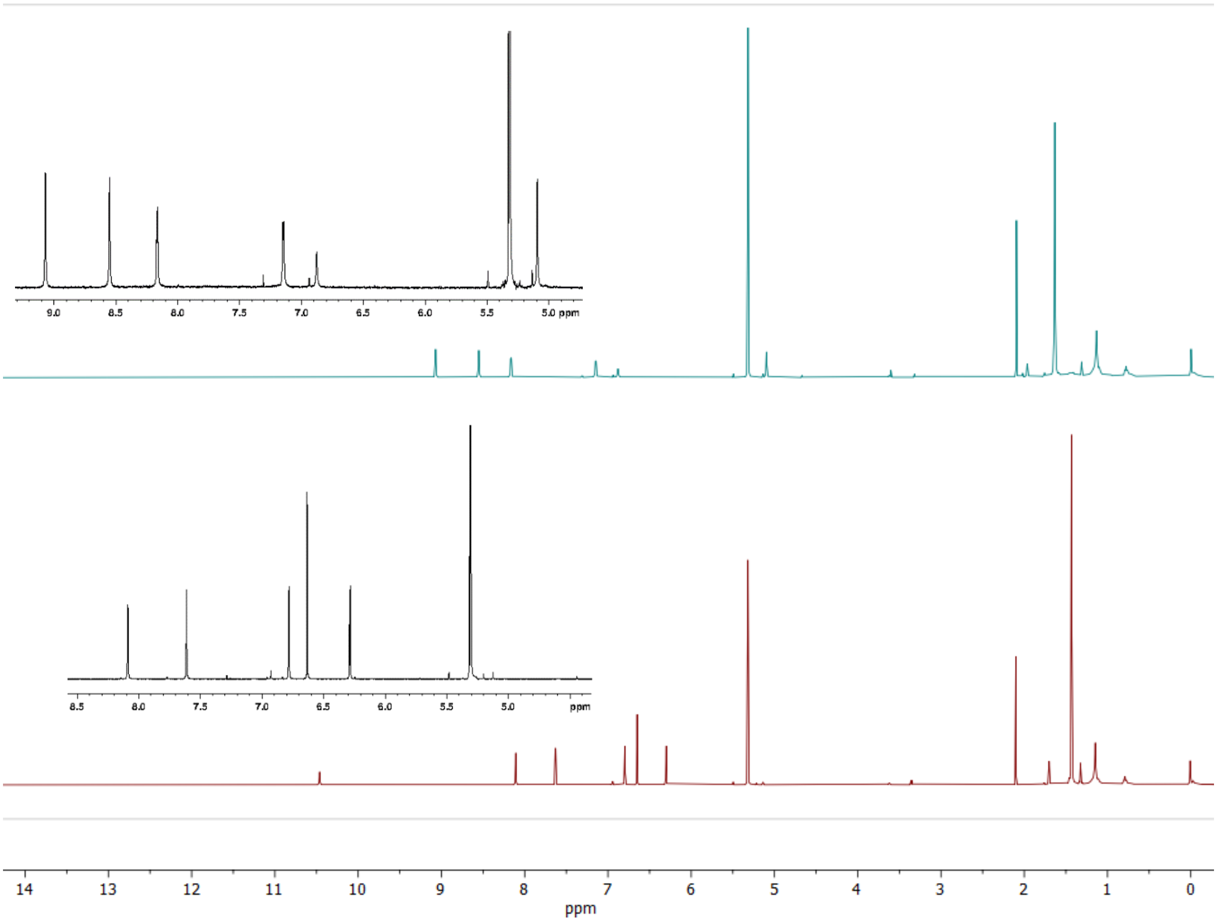

Figure S18. ^{1}H NMR-monitored titration of **5** and **5^{2+}** with $NOSbF_6$ (dichloromethane-d_2, 500 MHz, 193 K); red – without ; cyan – 2eq

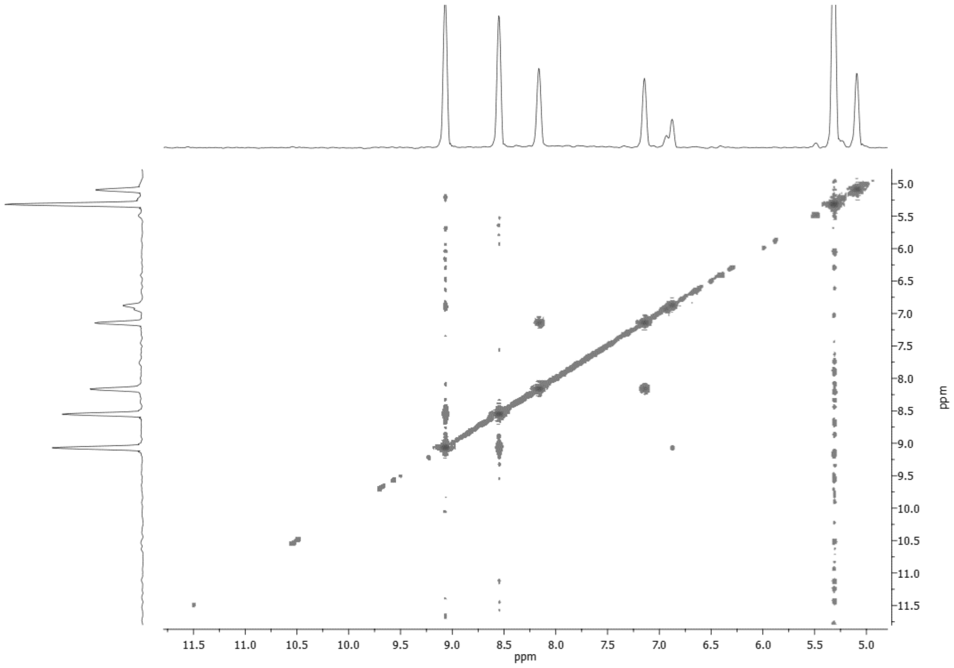

Figure S19. ^{1}H-^{1}H COSY NMR spectrum of **5^{2+}** (dichloromethane-d_2, 500 MHz, 193 K)

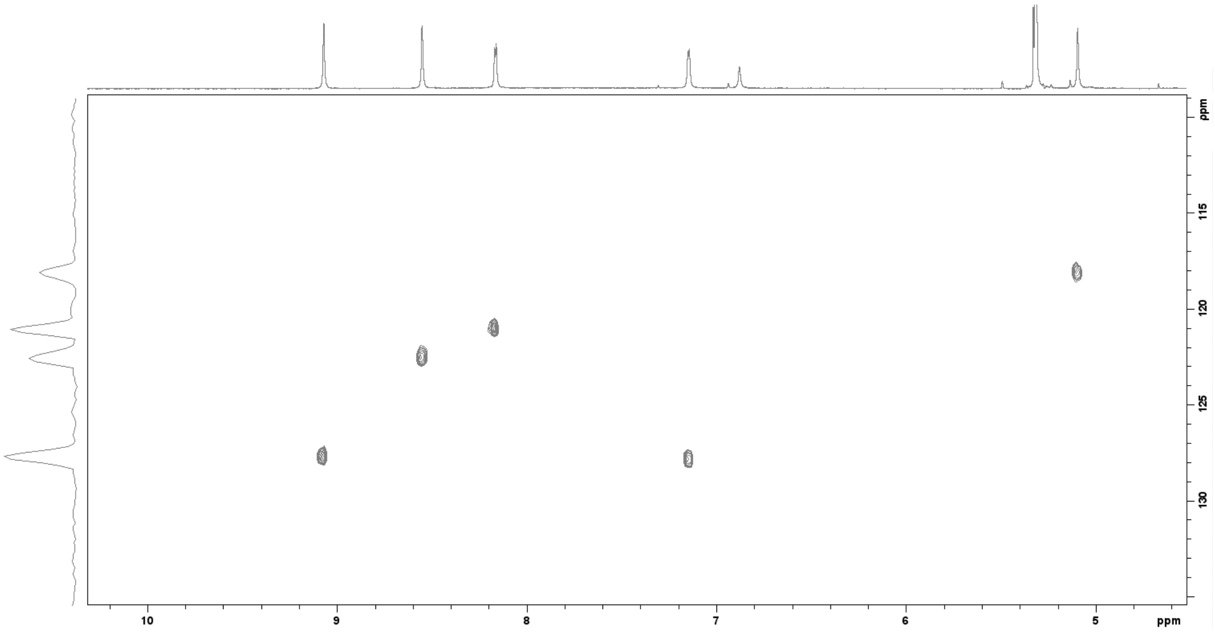

Figure S20. ^{1}H-^{13}C HSQC NMR spectrum of $\mathbf{5^{2+}}$ (dichloromethane-d_2, 500 MHz, 193 K)

4. UV-Vis experiments

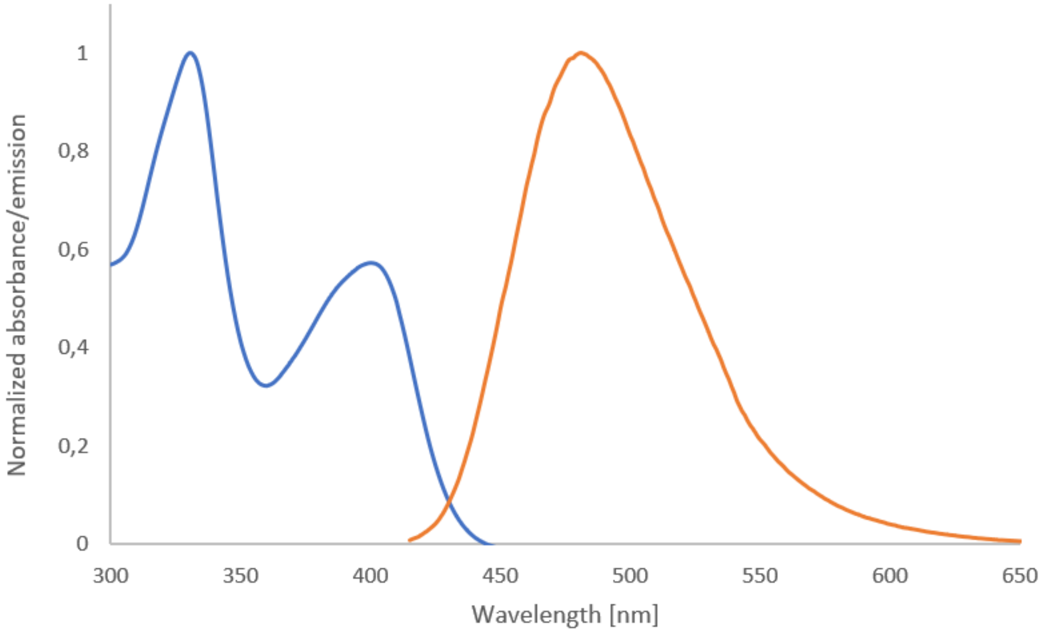

Figure S21. UV-Vis(blue line) and emission(orange line), spectra of **3** in acetonitrile at rt.

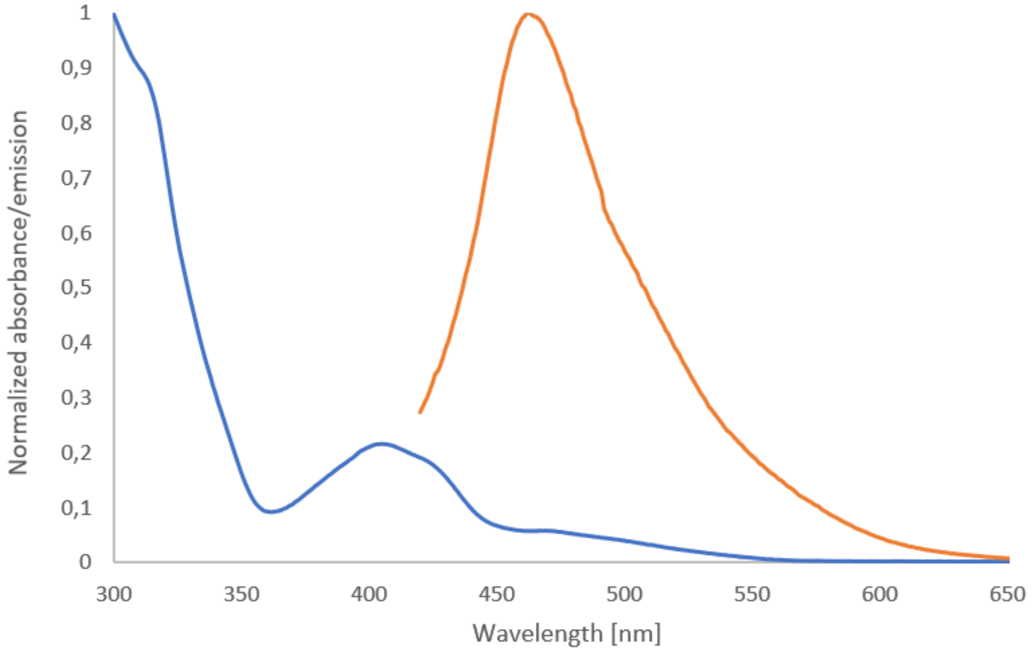

Figure S22. UV-Vis(blue line) and emission(orange line), spectra of **4** in acetonitrile at rt.

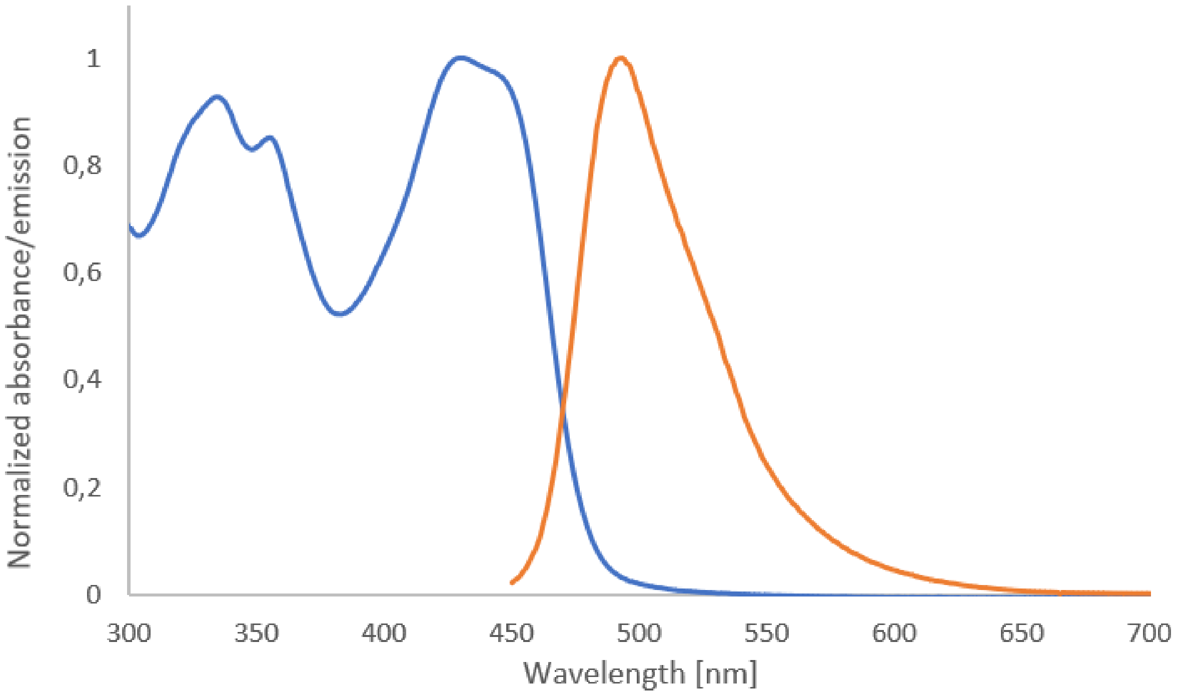

Figure S23. UV-Vis(blue line) and emission(orange line), spectra of **5** in acetonitrile at rt.

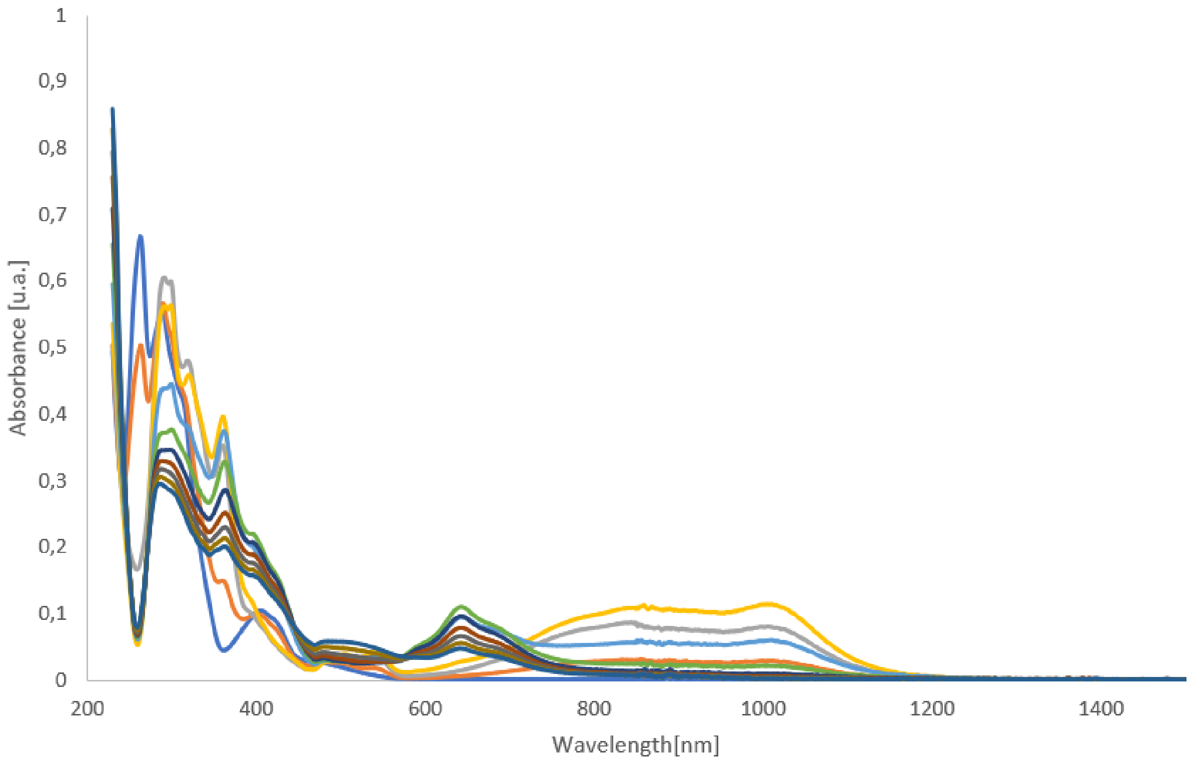

Figure S24. UV-Vis spectra recorded during titration of **4** to $\mathbf{4}^{2+}$ with $NOSbF_6$, from 0 to 2 equiv., inacetonitrile at rt.

5. X-Ray analysis

Identification code	**4**	**5**
CCDC Number	**2157938**	**2157939**
Empirical formula	$C_{30}H_{29}NO_2$	$C_{61}H_{60}Cl_2N_2O_4$
Formula weight	435.54	956.01
Temperature/K	100.15	100.00(10)
Crystal system	monoclinic	monoclinic
Space group	$P2_1/n$	$P2_1/c$
a/Å	13.20956(18)	16.2548(5)
b/Å	10.34483(13)	18.2091(4)
c/Å	16.3698(2)	17.8708(5)
α/°	90	90
β/°	94.9851(12)	105.832(4)
γ/°	90	90
Volume/$Å^3$	2228.48(5)	5088.8(3)
Z	4	4
ρcalc g/cm^3	1.298	1.248
μ/mm^{-1}	0.628	1.538
F(000)	928.0	2024.0
Radiation	CuKα (λ = 1.54184)	Cu Kα (λ = 1.54184)
2Θ range for data collection/°	8.258 to 151.76	7.072 to 109.996
Index ranges	-16 ≤ h ≤ 16<br>-12 ≤ k ≤ 12<br>-19 ≤ l ≤ 20	-17 ≤ h ≤ 17<br>-19 ≤ k ≤ 19<br>-18 ≤ l ≤ 18
Reflections collected	21868	9840
Independent reflections	4569<br>[R_{int} = 0.0209,<br>R_{sigma} = 0.0172]	9840<br>[R_{int} =<br>R_{sigma} = 0.0353]
Data/restraints/parameters	4569/0/308	9840/48/657
Goodness-of-fit	1.056	1.110
Final R indexes [I>=2σ (I)]	R_1 = 0.0398, wR_2 = 0.0996	R_1 = 0.1322, wR_2 = 0.3281
Final R indexes [all data]	R_1 = 0.0438, wR_2 = 0.1024	R_1 = 0.1542, wR_2 = 0.3440
Largest diff. peak/hole / e $Å^{-3}$	0.45/-0.40	0.92/-0.55

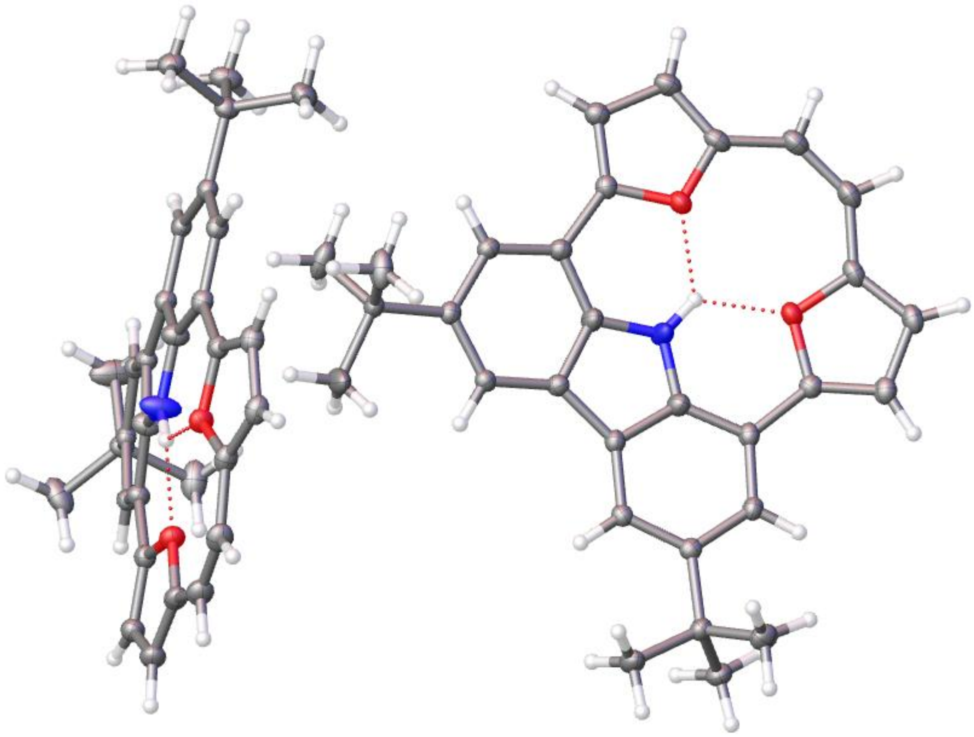

Figure S25. Crystal structure of **4**. Thermal ellipsoids present 50% probability.

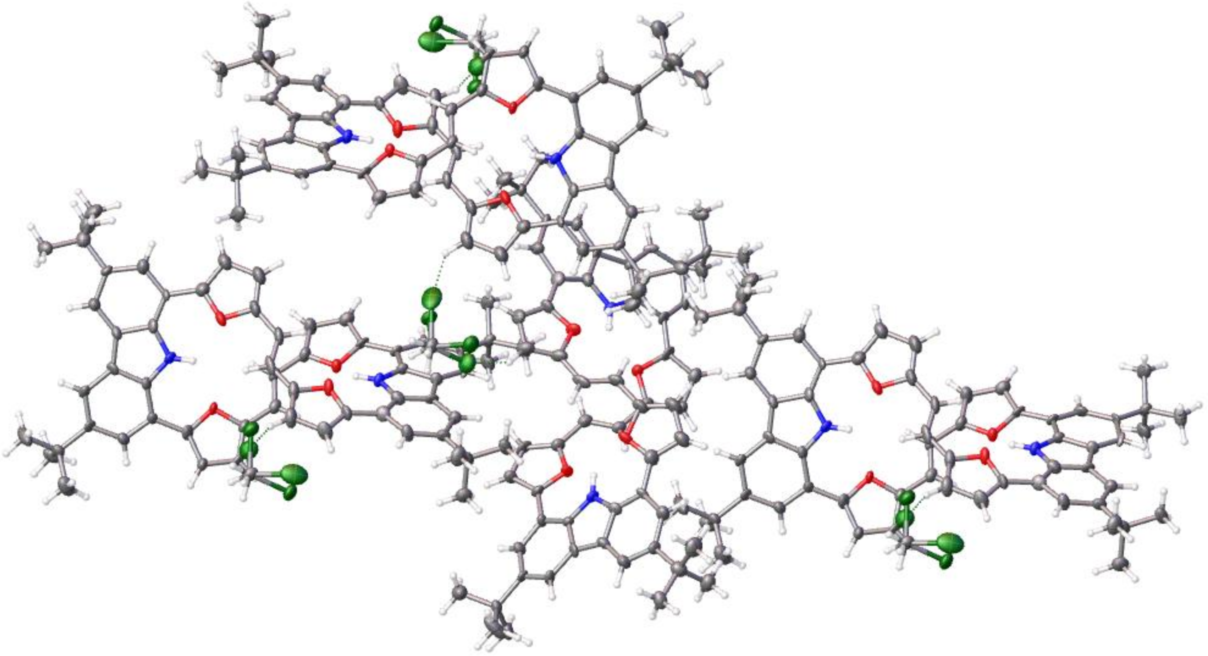

Figure S26. Crystal structure of **5**. Thermal ellipsoids present 50% probability.

6. Theoretical analysis

6.1. GIAO data

Table 1. GIAO chemical shifts vs. Experimental values

	**4**			**4^{2+}**			**5**			**5^{2+}**		
		Theory			Theory			Theory			Theory	
	Obs.	*	**	Obs.	*	**	Obs.	*	**	Obs.	*	**
NH	15.63	15.92	16.24	4.27	2.21	2.54	10.80	10.00	10.93	6.95	5.74	5.85
9^1,10^1	7.94	7.71	7.83	10.00	10.39	10.74	8.33	8.22	8.33	9.08	9.32	9.72
7^1, 12^1	7.47	7.48	7.41	9.72	10.32	10.47	7.76	7.98	7.95	8.59	9.04	9.20
5,14	6.76	6.54	6.52	10.19	10.24	10.46	6.49	6.89	6.91	8.18	8.02	8.34
4,15	6.45	6.20	6.22	10.09	10.15	10.39	5.97	6.40	6.38	7.19	6.85	7.03
1,2	5.88	5.29	5.22	9.86	10.00	10.20	6.72	6.66	6.89	5.10	5.28	4.68
*t*Bu	1.44	1.71	1.71	1.98	2.32	2.11	1.46	1.86	1.83			2.09
		1.69	1.14		2.23	2.14		1.81	1.26			1.82
		1.63	1.61		2.19	2.28		1.78	1.80			1.98
		1.57	1.18		2.06	2.28		1.74	1.79			1.81
		1.24	1.56		2.01	2.20		1.74	1.24			1.38
		1.24	1.14		1.95	2.04		1.34	1.32			1.80
		1.21	1.63		1.87	2.02		1.32	1.74			1.97
		1.19	1.22		1.81	2.15		1.24	1.30			1.92
		1.11	1.12		1.61	1.56			1.22			1.72

* B3LYP/6-31G(d,p) (values vs. TMS calculated at the same level of theory. Shielding constant 31.75);
** B3LYP/def2-TZVP/D3(BJ)/m5 (values vs. TMS calculated at the same level of theory. Shielding constant 31.93)

6.2. AICD plots

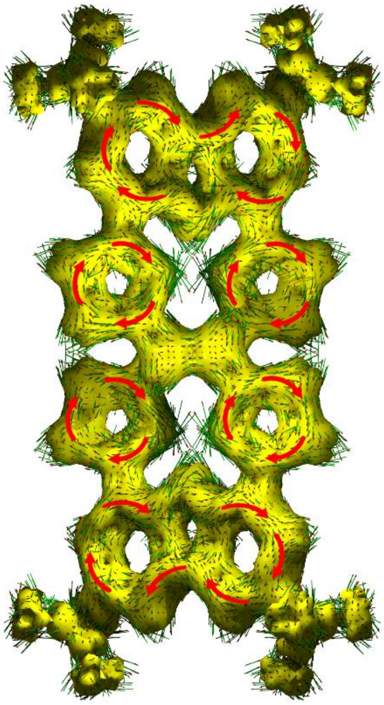

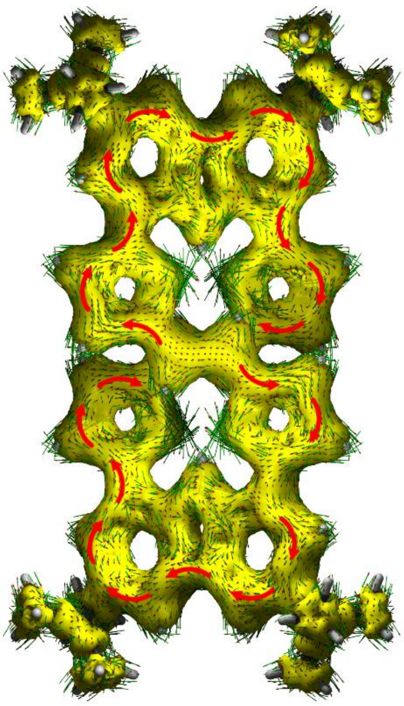

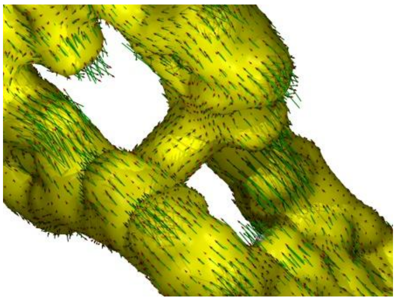

Figure S27. AICD plots for **5** (left) and $\mathbf{5^{2+}}$ (centre). Below the 3D through-space junction in $\mathbf{5^{2+}}$.

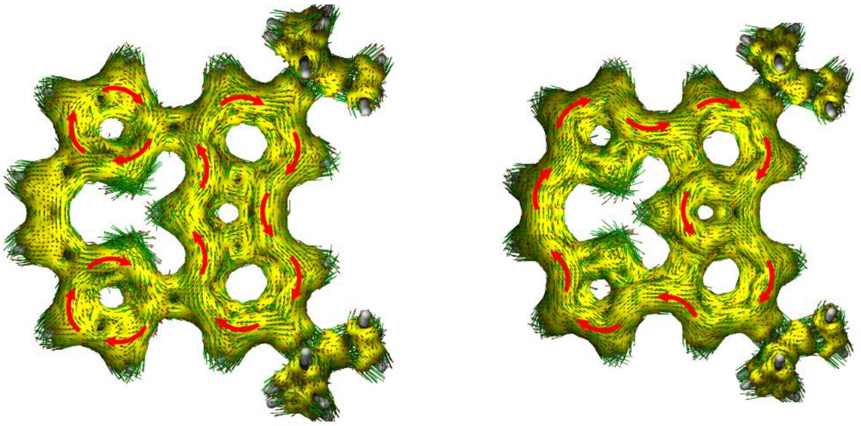

Figure S27. AICD plots for **4** (left) and $\mathbf{4^{2+}}$ (right).

6.3. GIMIC plots

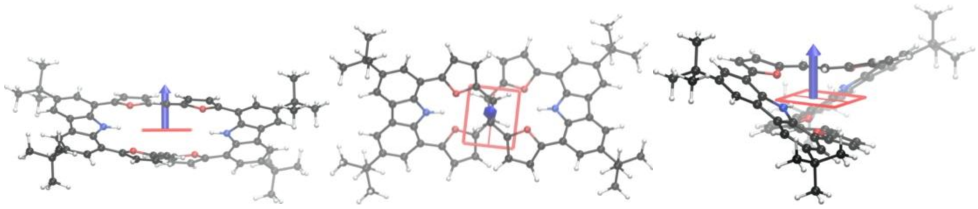

Figure S29. Illustration of the integration plane used to integrate the strength of the current-density flux in **5/5^{2+}** taking a through-space pathway parallel to the magnetic field vector, B$^{\perp}$, drawn in blue.

Analysing the magnetically induced current density: The analysis of the magnetically induced current density is done in two stages. First, the current density is calculated on a 3D grid and visualized with streamlines. This is accomplished by defining a set of points inside a sphere within the calculation grid. Then, the Runge-Kutta method[19,20] implemented in the PARAVIEW program[21] was employed to trace the trajectory of the current-density field. Current strengths in nA /T are obtained by integrating the current-density flux passing through a plane.

The neutral molecule sustains a fairly weak global diatropic ring current when the magnetic field is perpendicular to the figure-eight-shaped structure, B$^{\perp}$. The current-density pathways are shown in Figure S30.

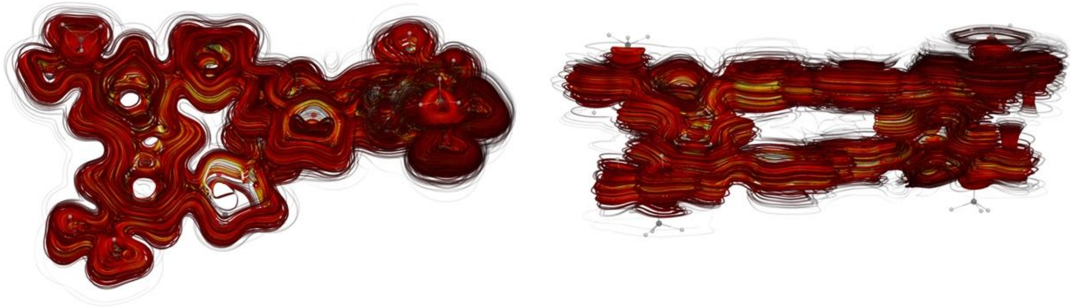

Figure S30. A streamline representation of the current density induced by B$^{\perp}$ in **5** from top (left) and from the side (right). The colour scheme represents the magnitude of the current density such that white is the strongest and black is the weakest.

[19] Runge, C. Über die numerische Auflösung von Differentialgleichungen. Math. Ann. 1895, 46, 167-178.
[20] Kutta, W. Beitrag zur näherungsweisen Integration totaler Differentialgleichungen. Z. Math. Phys. 1901, 46, 435-453
[21] Ahrens, J.; Geveci, B.; Law, C. ParaView: An End-User Tool for Large Data Visualization, Visualization Handbook, Elsevier, 2005, ISBN-13: 978-0123875822, see also: http://www.paraview.org

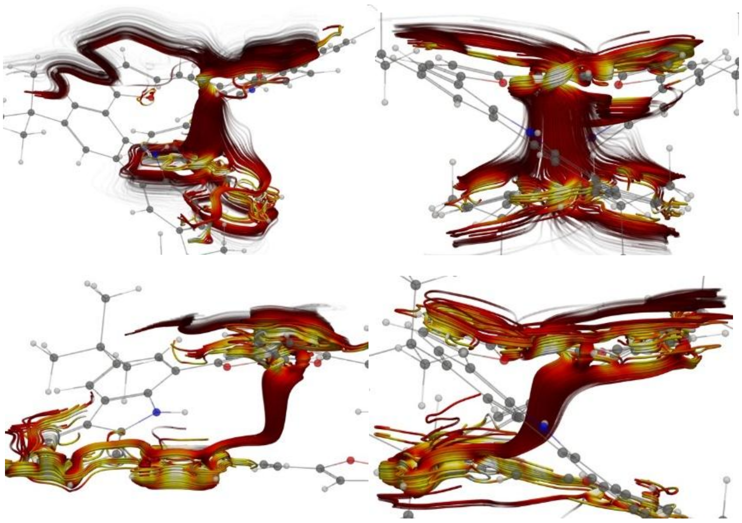

Figure S31. The through-space current density of the dication $\mathbf{5^{2+}}$ when the magnetic field is applied in the B$^{\perp}$ direction. The colour scheme represents the magnitude of the current density such that white is the strongest and black is the weakest.

The strength of the current density flowing vertically through the space between the two strands was determined by integration plane was positioned, so that it covers the whole current-density flux, when applying the magnetic field B$^{\perp}$, as illustrated in Figure S29. The strength of the vertical current-density flux that was observed only in the dication is 6.6 nA/T, which is about half the ring-current strength of benzene.[22] A map of the through-space current-density flux in the integration plane is shown in Figure S32.

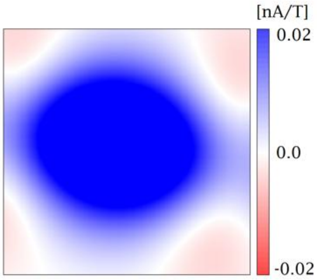

Figure S32. Map of the through-space current density flux passing through the integration plane between the two strands of $\mathbf{5^{2+}}$. The magnetic field is aligned in the B$^{\perp}$ direction.

An integration plane was placed to calculate the global ring-current strength, illustrated in Figure S33. It is obtained by taking vertical slices of the integration plane and integrating the current density in each of them, then plotting the results as a function of distance along the plane on the x axis. The integration starts inside the molecular structure, crosses the illustrated C=C bond and extends far out, where the current density vanishes. The sign is obtained by taking the scalar product of the current density with the normal of the integration plane, and thus the vectors pointing to the left or right have opposite signs. The obtained value of 5.2 nA/T suggests that the dication is weakly aromatic when the magnetic field is applied perpendicularly to the figure-eight structure (B$^{\perp}$). However, the integrated current-density flux consists of several pathways passing close to each other, and further rationalization is difficult. Some of the pathways are tracing the whole molecular structure, while others pass via the through-space shortcut. The corresponding ring-current profile is shown in Figure 5a, where the diatropic contribution is mainly outside the porphyrinoid ring and inside it the current-

[22] Fliegl, H.; Sundholm, D.; Taubert, S.; Jusélius, J.; Klopper, W. *J. Phys. Chem. A* **2009**, *113*, 8668-8676

density flux is paratropic as for porphyrinoids in general.[23,22] The ring-current strength obtained for the dication with the parallel orientation of the magnetic field ($B^{||}$) is 7.8 nA/T suggesting that it is almost as aromatic as benzene for this orientation of the magnetic field. There is no through-space current-density flux when the magnetic field is oriented in the $B^{||}$ direction.

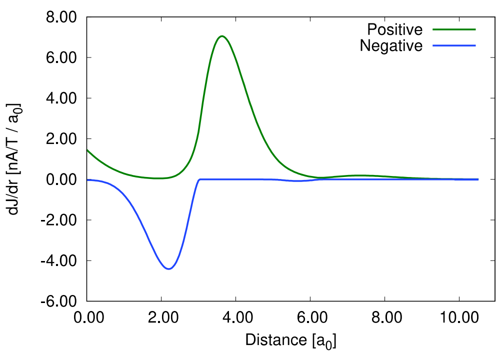

(a)

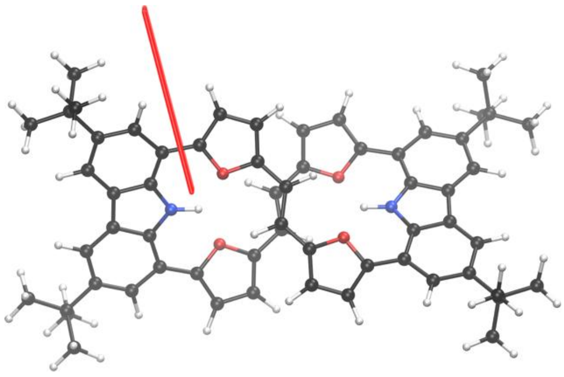

(b)

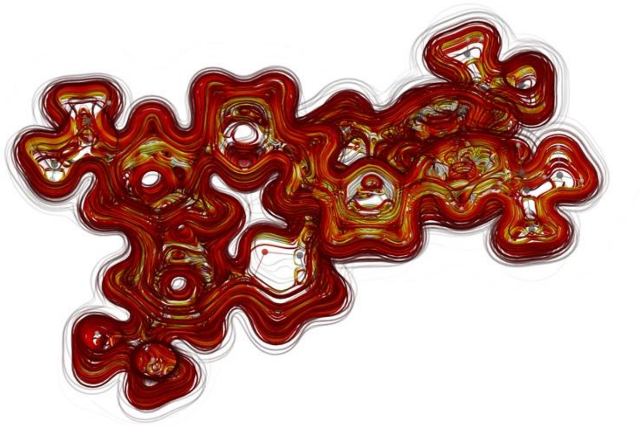

(c)

Figure S33. (a) The ring-current pro le of the dication obtained for $B^{\perp}$. (b) An illustration of the integration plane. (c) The corresponding current-density pathways are shown.

The aromatic character of the molecules can be understood from the circulation direction of the ring current with respect to the curl of the external magnetic field. Assuming that diatropic ring currents circulate in the clockwise direction in one half of the eight-shaped molecule and continues to the second half, it flows in the anti-clockwise direction in the other loop of the molecule. The conflict of the ring-current direction is the driving force for the shortcut current-density flux. Half of the molecule has a ring current in the aromatic direction and the other half would sustains a ring current in the antiaromatic direction even though it is globally aromatic. When the passage between the two strands is narrow in the middle of the molecule, the ring current prefers to make a shortcut instead of following the molecular framework to the other loop. When the distance between them is wide, the molecule becomes nonaromatic sustaining a very weak ring current around the whole molecular structure.

[23] H. Fliegl and D. Sundholm, *J. Org. Chem.* **2012**, *77*, 3408-3414.

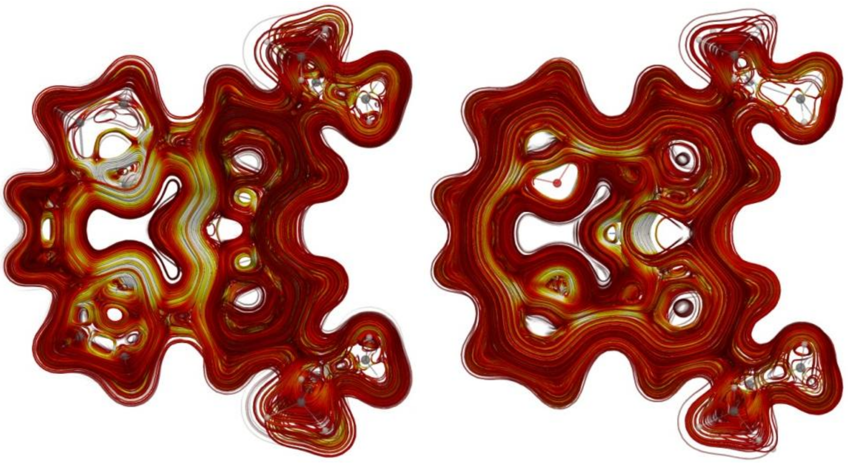

Figure S34. The current density of **4** (left) and $\mathbf{4^{2+}}$ (right).

4 has a strong paratropic ring current of about -9 nA/T that flows inside the whole molecule. The exact value is difficult to determine, because the diatropic and paratropic ring-current contributions are not conserved since bonds also sustain bond currents of different strengths. At the C2 double bond it is -11.4 nA/T, which is an upper bound. In Figure S34a, one can see a significant bond current in the C2 bond. In the formal single bond next to the C2 moiety, it is -9.6 nA/T. At the bond between the benzene ring and the furan ring, we get a value of -8.9 nA/T, which is a lower bound, because that integration is difficult to calculate accurately. A diatropic ring current of 9-10 nA/T flows around the carbazole moiety, since inside the pyrrole ring the paratropic contribution is -18.0 nA/T, which is the global paratropic ring current inside the molecule and the returning diatropic ring current around the carbazole. The whole molecule sustains a global diatropic ring current of about 6 nA/T, which is canceled by the stronger paratropic ring current leading to a net paratropic ring current and weak antiaromaticity. The local diatropic ring current around the furan rings is about 6 nA/T because the current strength along the outer pathway at the furan ring is 11.8 nA/T.

The current density shown in Figure S34b shows that $\mathbf{4^{2+}}$ is aromatic. It has a global ring current of 17.9 nA/T. The main part or 16.9 nA/T takes the outer route at the furan rings. The ring current splits at the carbazole moiety. At the pyrrole ring, the ring-current strength along the inner pathway is 11.1 nA/T and 7.1 nA/T flows along the outer one. However, the current density flux is even more complicated, because the ring-current strength on the outside of the benzene rings is 15.0 nA/T and only 2.9 nA/T takes the inner pathway. That means that the ring current flows between the benzene and the pyrrole ring from the outer pathway to the inner one. This is seen in Figure S34b. 42+ has a global paratropic ring current of -4.4 nA/T inside the molecule.

6.4. Cartesian coordinates

6.4.1. 4

N	1.1810641	-0.9987177	0.0000000
C	4.4714972	-0.2399178	1.7172438
C	-0.8831241	-0.4396976	-0.7331445
C	0.4519187	-0.7007742	1.1104981
C	2.4099668	-0.4340724	-2.5813729
C	-1.7945904	-0.1348833	-1.7475759
H	-2.8283466	0.0523493	-1.4977749
C	2.4099668	-0.4340724	2.5813729
C	0.4519187	-0.7007742	-1.1104981
C	4.4714972	-0.2399178	-1.7172438
C	-0.8831241	-0.4396976	0.7331445
C	0.9608811	-0.4805379	2.3966141
C	-1.3598335	-0.0291354	-3.0719169
C	3.2880396	-0.4092270	-3.6332803
H	3.0282117	-0.4784998	-4.6760297
C	5.4631283	-0.1475552	0.6820122
C	0.9608811	-0.4805379	-2.3966141
C	-1.3598335	-0.0291354	3.0719169
C	0.0121314	-0.1649103	3.3681412
H	0.3539381	0.0334349	4.3751381
C	0.0121314	-0.1649103	-3.3681412
H	0.3539381	0.0334349	-4.3751381
C	3.2880396	-0.4092270	3.6332803
H	3.0282117	-0.4784998	4.6760297
C	-1.7945904	-0.1348833	1.7475759
H	-2.8283466	0.0523493	1.4977749
C	5.4631283	-0.1475552	-0.6820122
C	4.5962822	-0.2834613	3.0882743
H	5.5220980	-0.2412654	3.6376127
C	4.5962822	-0.2834613	-3.0882743
H	5.5220980	-0.2412654	-3.6376127
C	-2.3241887	0.2989714	-4.2202349
C	-2.3241887	0.2989714	4.2202349
O	3.1465796	-0.3230019	-1.4222950
O	3.1465796	-0.3230019	1.4222950
H	6.4528150	-0.0669374	1.1184146
H	6.4528150	-0.0669374	-1.1184146
H	2.1794004	-0.9813599	0.0000000
C	-3.7785763	0.4109309	-3.7470078
C	-2.2539364	-0.8171741	-5.2796686
C	-1.9295926	1.6409276	-4.8659818
C	-3.7785763	0.4109309	3.7470078
C	-1.9295926	1.6409276	4.8659818
C	-2.2539364	-0.8171741	5.2796686
H	-3.9099934	1.2222082	3.0287014
H	-4.4245619	0.6198329	4.6015963
H	-4.1253823	-0.5159609	3.2865423
H	-2.9348021	-0.6007997	6.1064559
H	-1.2491647	-0.9188753	5.6911831
H	-2.5358578	-1.7785999	4.8462571
H	-0.9194689	1.6118712	5.2757382
H	-2.6135619	1.8855635	5.6822402
H	-1.9694508	2.4480153	4.1321084
H	-3.9099934	1.2222082	-3.0287014
H	-4.1253823	-0.5159609	-3.2865423
H	-4.4245619	0.6198329	-4.6015963
H	-2.5358578	-1.7785999	-4.8462571
H	-1.2491647	-0.9188753	-5.6911831
H	-2.9348021	-0.6007997	-6.1064559
H	-0.9194689	1.6118712	-5.2757382
H	-2.6135619	1.8855635	-5.6822402
H	-1.9694508	2.4480153	-4.1321084

Imaginary frequencies: 0
Total energy (*in hartree*) : -1365.6343110920

6.4.2. 4^{2+}

N	1.1426505	-0.8391893	0.0000000
C	4.4479718	-0.3229431	1.6958885
C	-0.8989104	-0.2941661	-0.7415194
C	0.4367816	-0.5882763	1.1110666
C	2.3851437	-0.4121164	-2.5669550
C	-1.7768272	-0.0217759	-1.7690988
H	-2.8126751	0.1980381	-1.5592110
C	2.3851437	-0.4121164	2.5669550
C	0.4367816	-0.5882763	-1.1110666
C	4.4479718	-0.3229431	-1.6958885
C	-0.8989104	-0.2941661	0.7415194
C	0.9735673	-0.4537417	2.4143950
C	-1.3295972	-0.0037416	-3.1298000
C	3.2877426	-0.2581108	-3.6375896
H	3.0211595	-0.1893188	-4.6793912
C	5.4191094	-0.2655308	0.7048432
C	0.9735673	-0.4537417	-2.4143950
C	-1.3295972	-0.0037416	3.1298000
C	0.0169364	-0.1921067	3.4225954
H	0.3505098	-0.0966962	4.4455430
C	0.0169364	-0.1921067	-3.4225954
H	0.3505098	-0.0966962	-4.4455430
C	3.2877426	-0.2581108	3.6375896
H	3.0211595	-0.1893188	4.6793912
C	-1.7768272	-0.0217759	1.7690988
H	-2.8126751	0.1980381	1.5592110
C	5.4191094	-0.2655308	-0.7048432
C	4.5604899	-0.2133989	3.1047463
H	5.4855550	-0.0944345	3.6462204
C	4.5604899	-0.2133989	-3.1047463
H	5.4855550	-0.0944345	-3.6462204
C	-2.3080444	0.2693271	-4.2753382
C	-2.3080444	0.2693271	4.2753382
O	3.1219086	-0.4583559	-1.4131380
O	3.1219086	-0.4583559	1.4131380
H	6.4124211	-0.1730827	1.1292447
H	6.4124211	-0.1730827	-1.1292447
H	2.1229130	-1.0482743	0.0000000
C	-3.7496628	0.4505330	-3.7843954
C	-2.2701001	-0.9267606	-5.2498982
C	-1.8772219	1.5547372	-5.0125069
C	-3.7496628	0.4505330	3.7843954
C	-1.8772219	1.5547372	5.0125069
C	-2.2701001	-0.9267606	5.2498982
H	-3.8569170	1.3165575	3.1280036
H	-4.3988755	0.6183876	4.6426412
H	-4.1227080	-0.4356801	3.2669145
H	-2.9659041	-0.7492785	6.0700589
H	-1.2801717	-1.0720080	5.6838871
H	-2.5638640	-1.8519354	4.7516058
H	-0.8832124	1.4641210	5.4519024
H	-2.5759613	1.7594772	5.8237602
H	-1.8781375	2.4147920	4.3408272
H	-3.8569170	1.3165575	-3.1280036
H	-4.1227080	-0.4356801	-3.2669145
H	-4.3988755	0.6183876	-4.6426412
H	-2.5638640	-1.8519354	-4.7516058
H	-1.2801717	-1.0720080	-5.6838871
H	-2.9659041	-0.7492785	-6.0700589
H	-0.8832124	1.4641210	-5.4519024
H	-2.5759613	1.7594772	-5.8237602
H	-1.8781375	2.4147920	-4.3408272

Imaginary frequencies: 0
Total energy (*in hartree*) : -1365.0745676360

6.4.3. 5

N	4.0861003	0.0000030	0.0000272
C	0.9568565	1.6377789	-1.7991134
C	6.2328917	-0.5879304	0.4264475
C	4.8776219	0.8987279	-0.6712492
C	3.0900738	-2.2762548	1.6851708
C	7.2285712	-1.4008972	0.9699725
H	8.2649819	-1.1628214	0.7812508
C	3.0900643	2.2762733	-1.6851222
C	4.8776275	-0.8987093	0.6713135
C	0.9568596	-1.6377671	1.7990263
C	6.2328884	0.5879819	-0.4263449
C	4.4938253	1.9957687	-1.4544785
C	6.8845869	-2.5096517	1.7411616
C	2.3475989	-3.4016658	1.9186046
H	2.7322435	-4.4039380	2.0023343
C	-0.0923634	0.6695331	-1.8012250
C	4.4938357	-1.9957455	1.4545492
C	6.8845746	2.5097181	-1.7410409
C	5.5239632	2.7811716	-1.9695393
H	5.2495794	3.6229792	-2.5910185
C	5.5239762	-2.7811144	1.9696567
H	5.2495965	-3.6228842	2.5911893
C	2.3475954	3.4016874	-1.9185596
H	2.7322369	4.4039672	-2.0022143
C	7.2285633	1.4009864	-0.9698220
H	8.2649732	1.1629720	-0.7810183
C	-0.0923638	-0.6695261	1.8010050
C	0.9854573	2.9970376	-1.9842839
H	0.1266131	3.6255696	-2.1501083
C	0.9854528	-2.9970232	1.9842201
H	0.1265984	-3.6255592	2.1499756
C	7.9366889	-3.4357534	2.3636224
C	7.9366753	3.4357426	-2.3636184
C	9.3669786	-3.0109411	2.0100344
H	9.5938603	-2.0088631	2.3785473
H	10.0766555	-3.7008891	2.4699357
H	9.5386596	-3.0281815	0.9321779
C	7.7254954	-4.8729318	1.8509865
H	6.7382613	-5.2540383	2.1142759
H	7.8206516	-4.9128151	0.7642282
H	8.4697831	-5.5447272	2.2851907
C	7.7975213	-3.4155832	3.8976881
H	6.8119275	-3.7550651	4.2178311
H	8.5425508	-4.0705764	4.3557353
H	7.9449333	-2.4056575	4.2848231
H	8.5424643	4.0704040	-4.3558054
C	7.7255492	4.8729640	-1.8510757
H	6.7383146	5.2540818	-2.1143466
H	7.8207569	4.9129222	-0.7643246
H	8.4698374	5.5447054	-2.2853623
C	9.3669685	3.0109124	-2.0100662
H	9.5386996	3.0282238	-0.9322187
H	9.5938034	2.0088016	-2.3785188
H	10.0766450	3.7008066	-2.4700489
C	7.7974359	3.4154667	-3.8976765
H	6.8118378	3.7549558	-4.2177983
H	7.9447990	2.4055087	-4.2847458
H	-1.0967693	1.0737869	-1.8249101
H	-1.0967739	-1.0737826	1.8244807
H	3.0836258	-0.0000031	0.0000232
N	-4.0861003	-0.0000037	0.0000363
C	-0.9568549	-1.6377761	-1.7991068
C	-6.2328923	0.5879304	0.4264525
C	-4.8776209	-0.8987285	-0.6712411
C	-3.0900766	2.2762609	1.6851739
C	-7.2285728	1.4008984	0.9699741
H	-8.2649831	1.1628221	0.7812512
C	-3.0900618	-2.2762727	-1.6851128
C	-4.8776285	0.8987104	0.6713194
C	-0.9568607	1.6377774	1.7990252
C	-6.2328877	-0.5879830	-0.4263384
C	-4.4938230	-1.9957692	-1.4544698
C	-6.8845898	2.5096547	1.7411612
C	-2.3476024	3.4016748	1.9185970
H	-2.7322457	4.4039499	2.0022977
C	0.0923646	-0.6695295	-1.8012203
C	-4.4938380	1.9957490	1.4545526
C	-6.8845718	-2.5097201	-1.7410342
C	-5.5239600	-2.7811725	-1.9695321
H	-5.2495753	-3.6229787	-2.5910128
C	-5.5239795	2.7811202	1.9696548
H	-5.2496009	3.6228949	2.5911810
C	-2.3475921	-3.4016857	-1.9185529
H	-2.7322348	-4.4039634	-2.0022266
C	-7.2285617	-1.4009879	-0.9698166
H	-8.2649719	-1.1629733	-0.7810147
C	0.0923634	0.6695373	1.8010051
C	-0.9854532	-2.9970366	-1.9842637
H	-0.1266063	-3.6255713	-2.1500633
C	-0.9854571	2.9970320	1.9842293
H	-0.1266062	3.6255656	2.1500118
C	-7.9366930	3.4357522	2.3636261
C	-7.9366715	-3.4357471	-2.3636098
C	-7.7255068	4.8729315	1.8509900
H	-7.8206672	4.9128150	0.7642320
H	-8.4697953	5.5447240	2.2851972
H	-6.7382731	5.2540417	2.1142757
C	-7.7975197	3.4155818	3.8976913
H	-7.9449262	2.4056553	4.2848263
H	-6.8119261	3.7550675	4.2178309
H	-8.5425503	4.0705717	4.3557414
C	-9.3669823	3.0109346	2.0100429
H	-9.5938590	2.0088558	2.3785567
H	-10.0766602	3.7008800	2.4699464
H	-9.5386669	3.0281742	0.9321869
C	-7.7255441	-4.8729673	-1.8510641
H	-6.7383094	-5.2540850	-2.1143347
H	-7.8207513	-4.9129234	-0.7643129
H	-8.4698320	-5.5447102	-2.2853492
C	-9.3669651	-3.0109175	-2.0100586
H	-9.5386960	-3.0282264	-0.9322110
H	-9.5938012	-2.0088079	-2.3785136
H	-10.0766409	-3.7008137	-2.4700394
C	-7.7974320	-3.4154741	-3.8976678
H	-6.8118335	-3.7549627	-4.2177889
H	-8.5424597	-4.0704131	-4.3557955
H	-7.9447962	-2.4055170	-4.2847392
H	1.0967707	-1.0737832	-1.8248989
H	1.0967734	1.0737942	1.8244800
H	-3.0836258	0.0000002	0.0000366
O	2.2406994	-1.1979833	1.6120156
O	2.2406915	1.1979916	-1.6120668
O	-2.2406906	-1.1979893	-1.6120631
O	-2.2406997	1.1979917	1.6120145

Imaginary frequencies: 0
Total energy (*in hartree*) : -2731.3415381830

6.4.4. 5^{2+}

N	-4.0749425	0.0000005	0.0000011
C	-0.9332387	1.6309010	1.7182101
C	-6.2111079	-0.6052363	-0.4095014
C	-4.8518945	0.9133298	0.6506851
C	-3.0677843	-2.2740908	-1.6633952
C	-7.1862275	-1.4445817	-0.9203354
H	-8.2279327	-1.2215485	-0.7457544
C	-3.0677849	2.2740924	1.6633975
C	-4.8518943	-0.9133300	-0.6506812
C	-0.9332387	-1.6308975	-1.7182093
C	-6.2111081	0.6052342	0.4095073
C	-4.4496433	2.0329840	1.4040650
C	-6.8304346	-2.5957769	-1.6556495
C	-2.3128542	-3.3829616	-2.0252484
H	-2.6997034	-4.3665298	-2.2312063
C	0.0971630	0.6817874	1.6880145
C	-4.4496428	-2.0329838	-1.4040614
C	-6.8304355	2.5957741	1.6556561
C	-5.4814543	2.8659541	1.8748352
H	-5.2066895	3.7302332	2.4625747
C	-5.4814533	-2.8659552	-1.8748304
H	-5.2066882	-3.7302336	-2.4625709
C	-2.3128557	3.3829638	2.0252510
H	-2.6997055	4.3665319	2.2312080
C	-7.1862280	1.4445781	0.9203432
H	-8.2279332	1.2215429	0.7457647
C	0.0971626	-0.6817834	-1.6880148
C	-0.9724667	2.9877605	2.0342354
H	-0.1175576	3.5976590	2.2753995
C	-0.9724655	-2.9877574	-2.0342326
H	-0.1175559	-3.5976558	-2.2753949
C	-7.8864279	-3.5445832	-2.2329970
C	-7.8864290	3.5445815	2.2330012
C	-9.3133148	-3.1143653	-1.8720649
H	-9.5587212	-2.1303913	-2.2762032
H	-10.0217215	-3.8247926	-2.2976276
H	-9.4751819	-3.1001705	-0.7924331
C	-7.6526589	-4.9623140	-1.6758773
H	-6.6722653	-5.3532821	-1.9510647
H	-7.7292135	-4.9730837	-0.5872302
H	-8.4030094	-5.6458950	-2.0751336
C	-7.7590300	-3.5650418	-3.7690346
H	-6.7795725	-3.9198904	-4.0922848
H	-8.5095698	-4.2323538	-4.1947663
H	-7.9137441	-2.5693044	-4.1880459
H	-8.5095714	4.2323567	4.1947689
C	-7.6526600	4.9623108	1.6758780
H	-6.6722661	5.3532793	1.9510640
H	-7.7292152	4.9730779	0.5872309
H	-8.4030101	5.6458931	2.0751331
C	-9.3133159	3.1143626	1.8720700
H	-9.4751826	3.1001648	0.7924383
H	-9.5587224	2.1303897	2.2762110
H	-10.0217226	3.8247911	2.2976306
C	-7.7590313	3.5650440	3.7690389
H	-6.7795739	3.9198938	4.0922882
H	-7.9137450	2.5693075	4.1880525
H	1.1033267	1.0753402	1.7566286
H	1.1033264	-1.0753362	-1.7566286
H	-3.0715724	0.0000013	0.0000000
N	4.0749424	0.0000006	-0.0000002
C	0.9332384	-1.6308994	1.7182065
C	6.2111080	0.6052348	-0.4095055
C	4.8518942	-0.9133305	0.6506813
C	3.0677848	2.2740942	-1.6633943
C	7.1862279	1.4445793	-0.9203404
H	8.2279331	1.2215438	-0.7457622
C	3.0677840	-2.2740925	1.6633930
C	4.8518944	0.9133304	-0.6506834
C	0.9332386	1.6309028	-1.7182083
C	6.2111078	-0.6052364	0.4095021
C	4.4496426	-2.0329852	1.4040602
C	6.8304356	2.5957759	-1.6556524
C	2.3128556	3.3829659	-2.0252469
H	2.6997055	4.3665340	-2.2312036
C	-0.0971629	-0.6817852	1.6880123
C	4.4496433	2.0329853	-1.4040623
C	6.8304344	-2.5957783	1.6556483
C	5.4814531	-2.8659566	1.8748292
H	5.2066880	-3.7302346	2.4625702
C	5.4814544	2.8659557	-1.8748320
H	5.2066896	3.7302340	-2.4625728
C	2.3128537	-3.3829638	2.0252447
H	2.6997028	-4.3665319	2.2312026
C	7.1862273	-1.4445822	0.9203357
H	8.2279326	-1.2215486	0.7457553
C	-0.0971632	0.6817892	-1.6880137
C	0.9724649	-2.9877597	2.0342281
H	0.1175551	-3.5976584	2.2753886
C	0.9724666	2.9877627	-2.0342316
H	0.1175575	3.5976617	-2.2753944
C	7.8864293	3.5445830	-2.2329978
C	7.8864276	-3.5445864	2.2329929
C	7.6526608	4.9623126	-1.6758752
H	7.7292158	4.9730801	-0.5872282
H	8.4030111	5.6458944	-2.0751305
H	6.6722671	5.3532814	-1.9510615
C	7.7590315	3.5650449	-3.7690355
H	7.9137453	2.5693082	-4.1880487
H	6.7795742	3.9198945	-4.0922849
H	8.5095716	4.2323574	-4.1947657
C	9.3133160	3.1143639	-1.8720666
H	9.5587223	2.1303908	-2.2762073
H	10.0217229	3.8247921	-2.2976274
H	9.4751829	3.1001663	-0.7924348
C	7.6526576	-4.9623157	1.6758701
H	6.6722639	-5.3532840	1.9510572
H	7.7292115	-4.9730829	0.5872230
H	8.4030079	-5.6458981	2.0751244
C	9.3133145	-3.1143683	1.8720608
H	9.4751805	-3.1001707	0.7924289
H	9.5587218	-2.1303955	2.2762015
H	10.0217212	-3.8247971	2.2976209
C	7.7590309	-3.5650485	3.7690306
H	6.7795734	-3.9198975	4.0922807
H	8.5095708	-4.2323618	4.1947602
H	7.9137457	-2.5693121	4.1880441
H	-1.1033268	-1.0753381	1.7566229
H	-1.1033270	1.0753423	-1.7566248
H	3.0715724	0.0000015	0.0000010
O	-2.2173632	-1.2139058	-1.4807070
O	-2.2173629	1.2139086	1.4807076
O	2.2173630	-1.2139073	1.4807057
O	2.2173628	1.2139100	-1.4807064

Imaginary frequencies: 0
Total energy (*in hartree*) : -2730.8410517350
